# Stochastic Cellular Automata Modeling of CO_2_ Hydrate Growth and Morphology

**DOI:** 10.1021/acs.cgd.3c00045

**Published:** 2023-05-19

**Authors:** Miguel Pineda, Anh Phan, Carolyn Ann Koh, Alberto Striolo, Michail Stamatakis

**Affiliations:** † Thomas Young Centre and Department of Chemical Engineering, University College London, Roberts Building, Torrington Place, London WC1E 7JE, United Kingdom; ‡ Institute for Materials Discovery, University College London, WC1H 0AJ, London, United Kingdom; ¶ Department of Chemical and Process Engineering, University of Surrey, Guildford, GU2 7XH, United Kingdom; § Center for Hydrate Research, Chemical & Biological Engineering Department, Colorado School of Mines, Golden, Colorado 80401, United States; ∥ School of Chemical, Biological and Materials Engineering, University of Oklahoma, Norman, Oklahoma 73019, United States

## Abstract

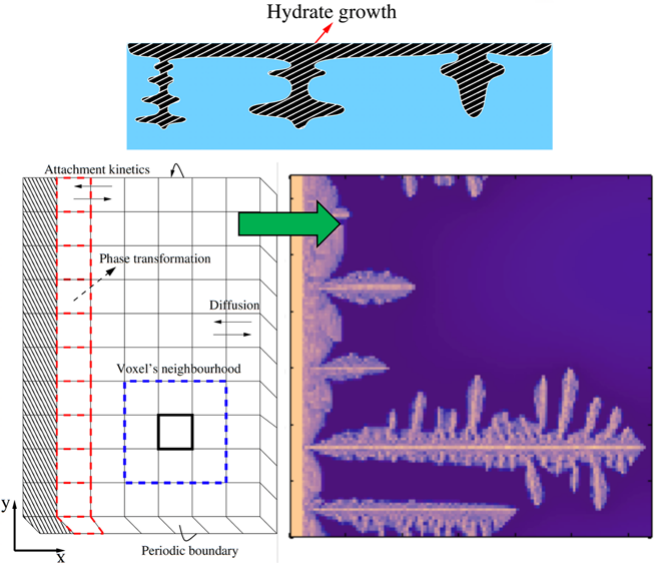

Carbon dioxide (CO_2_) hydrates are important in a diverse range of applications and technologies in the environmental and energy fields. The development of such technologies relies on fundamental understanding, which necessitates not only experimental but also computational studies of the growth behavior of CO_2_ hydrates and the factors affecting their crystal morphology. As experimental observations show that the morphology of CO_2_ hydrate particles differs depending on growth conditions, a detailed understanding of the relation between the hydrate structure and growth conditions would be helpful. To this end, this work adopts a modeling approach based on hybrid probabilistic cellular automata to investigate variations in CO_2_ hydrate crystal morphology during hydrate growth from stagnant liquid water presaturated with CO_2_. The model, which uses free energy density profiles as inputs, correlates the variations in growth morphology to the system subcooling *ΔT*, i.e., the temperature deficiency from the triple CO_2_–hydrate–water equilibrium temperature under a given pressure, and properties of the growing hydrate-water interface, such as surface tension and curvature. The model predicts that when *ΔT* is large, parabolic needle-like or dendrite crystals emerge from planar fronts that deform and lose stability. In agreement with chemical diffusion-limited growth, the position of such planar fronts versus time follows a power law. In contrast, the tips of the emerging parabolic crystals steadily grow in proportion to time. The modeling framework is computationally fast and produces complex growth morphology phenomena under diffusion-controlled growth from simple, easy-to-implement rules, opening the way for employing it in multiscale modeling of gas hydrates.

## Abstract 2

A physical-based computational modeling framework combining the Monte Carlo (MC) and cellular automata (CA) techniques provides insight into CO_2_ hydrate crystal growth and morphology.

## Introduction

Gas hydrates are solid structures formed at high pressure and low-temperature conditions from a solution of gas into water.^1,2^ Such structures, which are classified as structure I (sI), structure II (sII), and structure H (sH), consist of large and small cages of hydrogen-bonded water molecules that encapsulate the gas molecules. Hydrate-forming gas species include, for instance, xenon (Xe), carbon dioxide (CO_2_), and hydrocarbon molecules, such as ethane (C_2_H_6_), propane (C_3_H_8_), cyclopentane (C_5_H_10_), and methane (CH_4_). The type of structure formed depends primarily on the size of the gas molecule. Small gases such as CH_4_ and CO_2_ form sI hydrate structures, whereas large molecules such as C_3_H_8_ form sII hydrate structures. Gas hydrates with even larger molecules like neohexane (C_6_H_14_) together with a small one like CH_4_ form sH hydrates.^3^ Naturally occurring gas hydrates exist in sediments along the continental margins, deep lakes, and permafrost regions and are typically sI.^4^

Due to their unique properties, including a large gas storage capacity, a large heat of formation and dissociation, and gas selectivity, gas hydrates have several possible industrial applications,^7,8^ such as gas capture and storage,^9^ refrigeration systems,^10^ and water desalination.^11^ Because of these applications, basic and applied research on CO_2_ hydrate has been a topic of intense investigation. For instance, Zhang et al.^12^ reported that an optimal amount of CO_2_ liquefaction promotes CO_2_ hydrate formation on porous media. Similarly, CO_2_ capture micromechanisms and microstructures of stored forms together with hydrate growth rates were investigated by Zhang et al.^13^ In their work, based on the large amount of dissolved CO_2_ gas in water compared to other investigated gases, a mechanism for hydrate growth was proposed to promote CO_2_ capture and storage. Recent advances in CO_2_ sequestration and storage via gas hydrates have been recently summarized in a review article by Zheng et al.^14^ In particular, various approaches of CO_2_ sequestration via gas hydrates and their technical feasibility and potential storage capacity were discussed in that review. These approaches included storage in seawater, sediments under the sea floor,^15^ permafrost regions, and CH_4_ hydrate reservoirs via CO_2_–CH_4_ exchange. However, to further develop hydrate-based technologies, it is imperative to investigate the growth behavior of gas hydrates and the main factors affecting their morphology.^5^ Understanding hydrate crystal growth dynamics and morphology is necessary for instance in selecting their efficient formation method. Morphological studies could also deliver insights into the development of either innovative hydrate management alternatives (kinetic inhibitors and antiagglomerates) to mitigate the well-known oil and gas pipeline blockage due to internal hydrate-plug formation,^16^ or hydrate promoters.^17^ Concerning pipeline blockage, gas hydrate formation occurs as an interfacial phenomenon in which wettability and hydrate film growth and morphology within the pipeline dictate how easily the system may agglomerate due to capillary water bridging and sintering.^18,19^ Thus, this work aims to study gas hydrate crystal growth and morphology computationally.

Morphological studies of gas hydrates involve investigating their characteristic appearance, such as the size, shape, and mode of growth.^5^ In this respect, several experimental studies based on visual observations have revealed some features of the crystal growth dynamics and morphology of CO_2_ hydrates^20−23^ and hydrocarbon gas hydrates.^6,24−28^ For example, crystal growth dynamics and morphology of hydrates were found to depend on the nature of the gas (i.e., hydrate-former) molecules and the thermodynamic conditions of crystal formation. The subcooling temperature Δ*T* has been used as the driving force for hydrate crystal growth in such experimental works. Δ*T* is the difference between the experimental temperature *T*_ex_ of crystal formation and the triple gas-hydrate-water equilibrium temperature *T*_eq_ under a given pressure (Δ*T* = *T*_eq_ – *T*_ex_). In almost all these experimental studies, the relation between Δ*T* and the crystal growth morphology of several hydrate structures was revealed.^5^

**Figure 1:**
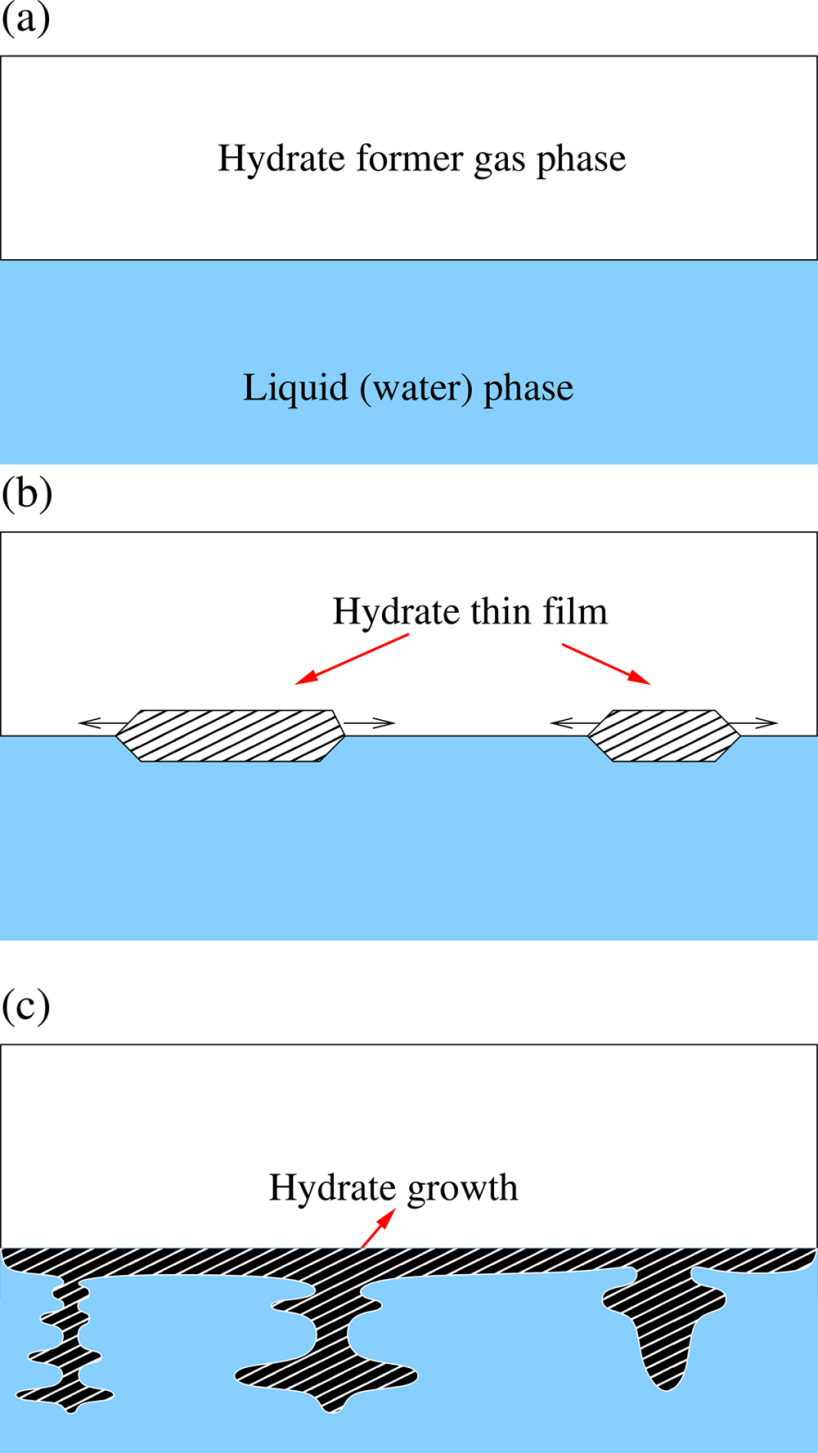
Schematic of gas hydrate growth in a batch gas–liquid water system.^5,6^ (a) Hydrate-former gas and water phases in equilibrium and separated by a flat interface. The concentration of hydrate-former in water in equilibrium with gas defines the solubility of the hydrate-former in water. In this panel, the system temperature, *T*_ex_, is slightly above the gas-hydrate-water equilibrium temperature, *T*_eq_. (b) Growth of an initial hydrate film covering the gas–water interface. (c) After the hydrate film covers the interface, the hydrate grows toward the water phase. Because it is assumed that the hydrate film blocks mass transfer across the interface, the hydrate growth morphology is only dependent on the solubility of hydrate-former in water. *T*_ex_ is below *T*_eq_ in (b) and (c). In this work, we are modeling the scenario of panel (c).

A generic observation was that, as long as the hydrate-former molecules are initially in a gas state (see Figure 1a),^23,24^ hydrate crystals preferentially form at the gas–water interface and grow in the form of a thin film covering the interface (see Figure 1b). Furthermore,^23,24^ it is observed that hydrate crystals could grow in liquid water from the initial hydrate thin film, if the hydrate-former molecules had dissolved in liquid water to saturation, or a specific concentration lower than saturation, before the hydrate formation (see Figure 1c). Although such a two-stage process typically occurred throughout the entire range of subcooling covered in the experiments, the morphology of the hydrate crystal growing toward water depended very much on the degree of subcooling.^23,24^ In particular, the experimental studies demonstrated that the morphology of the hydrate crystals that grew in the water from the hydrate thin film changed from skeletal polyhedral or columnar to dendritic depending on Δ*T*.^23,24^ The mechanism behind this morphology change toward stagnant liquid water presaturated with a hydrate-forming substance has been qualitatively rationalized based on the idea that such a variation depends on the growth rate of the hydrate crystal, which in turn is controlled by the diffusion of the hydrate-former molecules dissolved in the bulk liquid-water to the hydrate thin film surface.^23−25,29,30^ This diffusion was proposed to be driven by the difference in hydrate-former (e.g., CO_2_ and CH_4_) concentration in liquid water adjacent to the hydrate thin film surface (i.e., the concentration of hydrate-former in liquid water in equilibrium with hydrate) and the hydrate-former concentration in the bulk liquid-water phase, given by the concentration in liquid water in equilibrium with gaseous hydrate-former (i.e., the solubility of hydrate-former in water). Such a difference in concentration increases with Δ*T*.

The CO_2_ hydrate formation kinetics and morphology resulting from the interaction between liquid CO_2_ and water has also been the subject of experimental investigations due to its potential impact on reducing CO_2_ emissions. The motivation behind those studies is the possibility of injecting these emissions as compressed liquid CO_2_ into the deep ocean sediments to store as gas hydrates.^20,21,31−34^ A typical observation is the occurrence of different stages of hydrate formation, namely, hydrate nucleation, lateral and thickening hydrate film formation at the interface between liquid CO_2_ and water, and hydrate growth. The CO_2_ hydrate crystallization and growth from an aqueous CO_2_ solution at pressures to 6.5 MPa have also been explored experimentally.^35^

Most empirical morphological studies have been performed in batch systems, where the water is stagnant and presaturated with the hydrate-former substance (see Figure 1). However, a few studies have explored the growth morphology of gas hydrate crystals under flow conditions.^36−38^ The crystal growth morphology of gas hydrates formed with multihydrate former gas mixtures has been reported.^26,39−42^ The morphology of methane hydrate formation in porous media is also documented.^43^ The morphological study of methane hydrate formation and dissociation in the presence of amino acids as kinetic promoters is also available in the literature.^44^ Significant morphological differences using amino acid compared to the commonly used kinetic promoter, sodium dodecyl sulfate (SDS) surfactant, were found at the same concentrations under similar experimental conditions. The effect of salts, such as NaCl, on hydrate formation morphology has been studied as well.^45,46^ The crystal growth morphology of structure-H hydrate has been experimentally explored.^28^ More recent studies have reported the crystal growth morphology of gas hydrates with ozone,^47^ CH_4_ + tetrahydropyran (THP) hydrates,^48^ and CO_2_ hydrate in aqueous fructose solution.^22^

Although the experimental studies of hydrate crystal growth morphology have achieved considerable progress during the last few decades,^5^ the physical mechanisms leading to the morphological transition of gas hydrate crystals growing from a hydrate thin film into liquid water as a function of thermodynamic conditions remain unclear. The lack of quantitative information from current visual experimental studies could be the reason for such a lack of understanding. This is where computational modeling could provide significant insight. However, such computational modeling studies of gas hydrate growth morphology are scarce in the literature.

Molecular dynamics (MD) simulations are the main computational tools for understanding the fundamental molecular mechanisms behind gas hydrate growth.^49−53^ However, MD simulations are limited to short simulation times and small domains and, therefore, cannot be used to perform large-scale computational studies of gas hydrate morphology. Yet, MD can be coupled with other mesoscopic theoretical frameworks, like the phase-field (PF) method, to model large-scale properties of gas hydrates.^54−59^ This approach has been adopted by Tegze et al.^56^ to predict the nucleation and growth rates of solid CO_2_ hydrate in CO_2_ aqueous solutions under conditions typical to oceanic natural gas hydrate reservoirs. Although PF theory is one of the most powerful methods that are used to describe solidification phenomena in undercooled liquids,^60^ simulations based on it are generally very computationally demanding as well.

Motivated by those computational challenges, Buanes et al.^61,62^ developed a simplified framework for simulating large-scale hydrate kinetics based on a Monte Carlo (MC) cellular automaton (CA) approach to evaluate the most probable growth path. They also applied it to hydrate growth from a supersaturated aqueous solution of CO_2_ under conditions typical to oceanic natural gas hydrate reservoirs. They concluded that the MC-CA approach has the benefit of being much more computationally efficient and is still giving results consistent with the PF theory. Although Buanes et al.^61,62^ did not conduct a detailed sensitivity analysis of CO_2_ hydrate growth morphology, the novelty of their work was combining the two numerical approaches to study gas hydrate kinetics.

The MC and CA modeling approaches are well-established mesoscopic procedures to simulate natural phenomena. In particular, CA was introduced by John von Neumann and Stan Ulam as a technique to model biological self-reproduction.^63^ Since then, this approach has attracted significant attention in computer science, biology, and other fields, and it is, in fact, a well-established mesoscopic modeling approach in materials science.^64−66^ A CA is a dynamic system composed of a lattice of cells. Each cell in the lattice can assume a state from a given set of states. The system evolves in time according to an updating rule which is a function of the cell state itself and the state of neighboring cells. All the cells are updated synchronously, and the updating rule can be either deterministic or probabilistic. A CA with probabilistic cell state change rules is called a probabilistic CA. A cellular automaton can also be combined with other computational methods to get what is known as hybrid CA modeling. The appealing features of the CA approach are its computational efficiency and its ability to capture complex real-world behavior from simple rules. In this respect, CA provides insights into the complex behavior of gas hydrate growth morphology and could even be fast enough to be implemented into flow modeling tools relevant to gas hydrate formation and flow assurance.^67^

In this work, we build on and extend the MC-CA-type modeling approach by Buanes et al.^61,62^ In particular, we implement a hybrid probabilistic CA modeling framework to explore qualitative and generic features of sI CO_2_ hydrate crystal growth morphology toward liquid water presaturated with CO_2_. Thus, we focus on a batch system where the morphological hydrate growth proceeds toward a stagnant water phase from a pre-existing hydrate film. We assess computationally the proposed mechanism by which the hydrate crystal growth morphology is caused by the diffusion of CO_2_ molecules to the hydrate film due to a CO_2_ concentration gradient in the aqueous solution. To this end, we correlate the growth morphological change to the magnitude of Δ*T* and properties of the growing hydrate-water interface, such as surface tension and curvature. Our findings could have implications for CO_2_ storage technologies, since CO_2_ hydrates have been suggested to be a stable storage form for this well-known greenhouse gas.^68−70^ However, the fate of the stored CO_2_ would strongly depend on the CO_2_ hydrate formation pathway. Therefore, it is imperative to investigate the morphology of CO_2_ hydrate crystals grown in well-defined systems and to know what factors control these morphological phenomena before moving toward more realistic hydrate conditions in the geological storage of CO_2_, where a continuous supply of CO_2_ is expected.

The rest of the paper is organized as follows. In the next section, we present the hybrid probabilistic CA modeling approach and provide details about its numerical implementation. The two last sections are devoted to simulation results, discussion, and conclusions.

## Modeling Approach

We study the crystallization front morphology of sI CO_2_ hydrate growing from a sI CO_2_ hydrate thin film toward quiescent water with dissolved CO_2_.^23^ Because it is assumed that this hydrate film limits mass transport of CO_2_ across the CO_2_-aqueous solution interface,^71^ the hydrate formation and growth will happen only due to the CO_2_ dissolved in water (see Figure 1). Furthermore, CO_2_ hydrate formation will occur at the hydrate-water interface, and hydrate formation in the bulk of the water solution will be neglected. The CO_2_ hydrate crystallization front morphology is explored as a function of the subcooling Δ*T* and hydrate-water interface properties.

**Figure 2:**
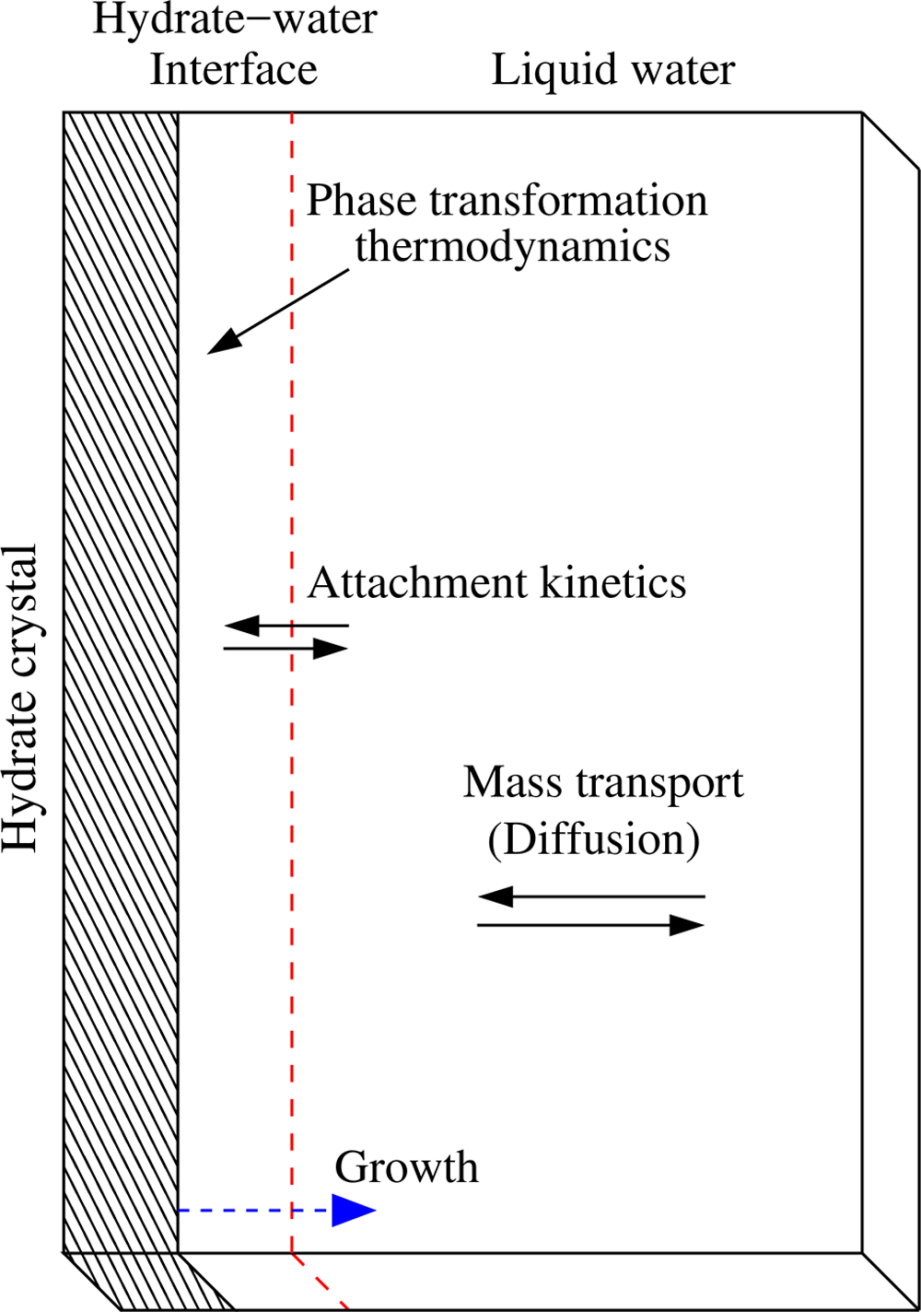
Schematic representation of the simulated domain, showing a sI CO_2_ hydrate crystal region led by a hydrate-water interface that propagates toward the stagnant water with dissolved CO_2_. This hydrate-water interface has a specific thickness. The modeling approach’s physical processes are (1) the thermodynamics of phase transformation between liquid state (water with dissolved CO_2_) and solid state (CO_2_ hydrate), (2) the interface attachment (or integration) kinetics of CO_2_ molecules, and (3) the mass transport by diffusion of CO_2_ molecules inside the stagnant liquid water phase.

As Figure 2 shows, the simulation domain of interest pertains to a sI CO_2_ hydrate crystal region led by a phase boundary (i.e., hydrate-water interface) that propagates in time toward the water with dissolved CO_2_. This interface is the region of volume where the transition between the solid hydrate state and liquid state occurs and can be considered as an integration (solidification) layer that consists of a number of water cages under formation. The modeling approach’s physical processes are (1) the thermodynamics of phase transformation between the two phases at the moving hydrate-water interface, (2) the interfacial attachment (or integration) kinetics of CO_2_ molecules at that interface, and (3) the diffusion of CO_2_ molecules inside the stagnant liquid water region.^72^ These processes are the centerpieces of this work, as we aim to simulate hydrate crystal growth and assess how the pertinent mechanisms give rise to morphological changes. The following sections describe how these processes are incorporated into the hybrid probabilistic CA framework implemented in this work. Following Ohmura et al.,^23,24^ the model does not consider the interface temperature increment due to heat release from the hydrate formation. This assumption is justified by the observation that the thermal transport of liquid-water and hydrate crystal is several orders of magnitude faster than the diffusion of CO_2_ in liquid water.^23,24,73^ Furthermore, CO_2_ diffusion along the hydrate crystal is several orders of magnitude slower than in the liquid water phase and, therefore, will also be neglected.^23,24,71^

With these aspects in mind, let us describe in the following section the computational simulation approach of this work.

### Hybrid Probabilistic Cellular Automaton Framework

We introduce in this section the hybrid probabilistic CA approach implemented in this work. We will define the CA lattice of cells associated with the simulation domain presented in Figure 2, the possible states of the cells, and the probabilistic updating rule of those states. The latter will be based on the MC method.^61,62^ The interaction between cells due to mass transport by diffusion is performed using a MC implementation of Fick’s law and the incorporation of CO_2_ to the integration layer defined by the interface is modeled by Fick’s law alone.^61,66^ These implementations of diffusion and incorporation are combined with the CA probabilistic updating rule, giving rise to this work’s hybrid probabilistic CA approach.

#### Cellular Automaton Lattice, State, and Composition

**Figure 3:**
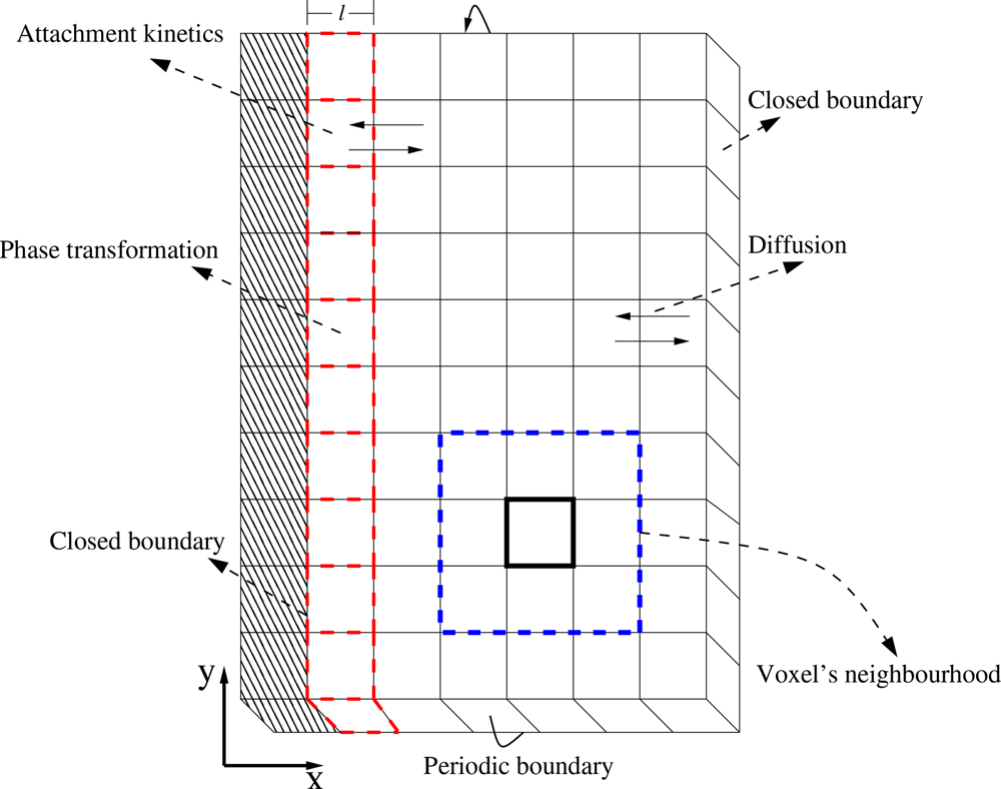
Schematic representation of the CA lattice associated with the simulation domain (see Figure 2). The CA lattice consists of cubic voxels of volume, *V* = *l*^3^, containing CO_2_ and water. There are periodic boundaries along the *y* axes and closed boundary conditions along the *x* direction. The interface is a moving boundary because their voxels, here represented in red dashed lines, can perform phase transformations between a liquid-like state (water with dissolved CO_2_) and a solid (or CO_2_ hydrate) state. The attachment kinetics and the diffusion mass transport between voxels are also highlighted. The bottom right of the figure shows a 2D representation of the voxel’s neighborhood. The side length of the whole simulation domain in the *x* direction will be denoted by *L*_*x*_ and the one for the *y* direction as *L*_*y*_. These two lengths are a multiple of the voxel side length *l*, whose size is chosen to be of the order of the interface thickness. Although not shown in the figure, the side length in the *z* direction is *L*_*z*_ = *l*. The voxels are characterized by their composition, given by a CO_2_ molar fraction of χ_c_ and a water molar fraction of χ_w_ = 1 – χ_c_, as defined in the main text.

As Figure 3 shows, the CA lattice associated with our simulation domain is constructed by dividing it into cubic cells or voxels of equal and fixed volume, *V* = *l*^3^, where *l* will be of the same order as the hydrate-water interface thickness.^61,62^ The voxels of the hydrate crystal are portrayed with diagonal hash patterns, and the ones belonging to the hydrate-water interface have their borders highlighted by red dashed lines. The rest of the voxels are presented with border-lines in black. The bottom right of the figure shows a 2D representation of the voxel’s neighborhood implemented in this work. It contains the so-called von Neumann neighborhood, in which a voxel is only connected to its first nearest neighbor (nn) voxels, and the Moore neighborhood, where the first and second nearest neighbors are included.^65^ As Figure 3 also shows, there are no domain boundaries along the *y* direction because we assume periodic boundary conditions. However, closed boundary conditions are considered along the *x* direction. The latter defines two boundaries; the first involves voxels on the hydrate-water interface. The voxels at the end of the domain along the *x* axes provide the second boundary. The requirement for a voxel to be in the hydrate-water interface is having at least a hydrate voxel nearest neighbor. Note, however, that this hydrate-water interface boundary is moving toward the water region because, its voxels are allowed to perform phase transformations between a liquid-like state (water with dissolved CO_2_) and a solid (or CO_2_ hydrate) state.

The voxels are not only characterized by their neighborhood but also their state, denoted here by the Greek letter ϕ. For liquid water voxels with dissolved CO_2_ (including the interface ones), ϕ takes the value of 0, and for a hydrate (i.e., solid) voxel, it takes the value of 1. The voxels are also characterized by their composition given by a CO_2_ molar fraction of χ_c_ = *n*_c_/(*n*_c_ + *n*_w_) and a water molar fraction of χ_w_ = 1 – χ_c_, with *n*_c_ and *n*_w_ been the moles of CO_2_ and water, respectively, inside the voxel.

With the CA lattice, neighborhood, as well as voxel state and composition defined, we are ready to introduce the evolution rules of the hybrid probabilistic CA simulation framework, which are based on the three physical processes mentioned above, namely, phase transformation, attachment kinetics, and mass transport by diffusion. From a computational viewpoint, starting at a time, *t*, equal 0, these rules are applied at each iteration (after time *Δt*), to update the states and compositions of the voxels. The pertinent iterative process is explained in detail later, together with the definition of *Δt*. Let us first explain each of the rules.

#### Hydrate Phase Transformation

Let us describe the protocol for the transition from liquid water with dissolved CO_2_ state and a CO_2_ solid hydrate state, which pertains to the interfacial voxels (i.e., the voxels having at least one hydrate voxel as their neighbor, depicted in red dashed lines in Figure 3). The remaining liquid water voxels do not perform a phase transformation because hydrate formation in the bulk liquid water is not considered. In other words, nucleation is neglected in the current approach. At each time step, *Δt*, all interfacial voxels are allowed to attempt a phase transformation between liquid and solid hydrate. The basis of such a phase transformation is the Glauber transition probability for an interfacial liquid voxel to become a solid one, given as^74,75^
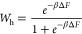
1and the one for the remelting,
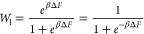
2where *W*_h_ + *W*_l_ = 1 (normalization), *W*_h_/*W*_l_ = *e*^–*βΔF*^ (detailed balance), and β = (*k*_B_*T*)^−1^, with *k*_B_ being the Boltzmann constant and *T* the temperature of the system, which is assumed to be constant during the simulation.^74^ Thus, if a uniform random number (uniform deviate) *r*_h_ is less than *W*_h_ solidification takes place; however, if another uniform deviate *r*_l_ is less than *W*_l_ the solidified voxel returns to its original liquid state.

Following Buanes et al.,^61,62^ we assume that *ΔF* = Φ(ϕ, λ)*Δf*(χ_c_, *T*), where *Δf*(χ_c_, *T*) is the change in free energy when an interfacial voxel with CO_2_ molar fraction χ_c_ and temperature *T* changes its phase from liquid to solid, and Φ(ϕ, λ) is a term accounting for the solid–liquid interface effects, like for example surface tension and interfacial curvature. Thus, to obtain *Δf*(χ_c_, *T*) = *f*_h_ – *f*_*l*_, we need expressions for the bulk free energy densities of the liquid water with dissolved CO_2_ (*f*_l_) and solid CO_2_ hydrate (*f*_h_) phases as a function of χ_c_ and *T*. This work will use the free energy density expressions implemented in the literature^54−57,76^ to obtain the equilibrium isobaric water-CO_2_ phase diagram describing the zones of CO_2_ hydrate stability in terms of temperature and composition. The free energy density for the solid hydrate state, *f*_h_, was constructed assuming a sI CO_2_ hydrate structure with large cages fully occupied by CO_2_ molecules and small ones empty. For further details regarding these free energy densities and the equilibrium phase diagram, refer to the Appendix.

The term Φ(ϕ, λ) defined as^61,62^
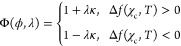
3accounts, in a qualitative way, for the role played by the local interface curvature κ on the probability of performing phase transformation. The strength of this curvature (or surface tension) effect is parametrized with the dimensionless parameter λ ≥ 0. To approximate the local interface curvature, we use the method of Liu and Goldenfeld,^61,62,66,77^ with the curvature expressed as
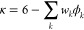
4where the summation index *k* is over the first and second nearest neighbor voxels of the interfacial voxel in question (Moore neighborhood), ϕ_*k*_ is their state (1 for solid hydrate and 0 for liquid), and *w*_*k*_ is a weighting term equal to 2 for first nearest neighbors and 1 for second nearest neighbors. The value of 6 corresponds to a summation on a flat interface (i.e., κ = 0) and is taken equal to one-half the sum over the entire weighted neighborhood with all voxels in a solid hydrate state. We refer to the Appendix for an explanation of how this value is obtained.

**Figure 4:**
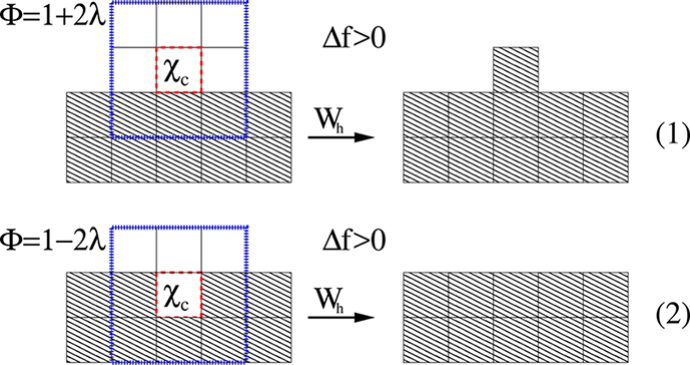
A liquid-to-solid transition as performed by the interfacial voxel in red dashed lines under two different neighborhood conditions. Because in both cases the voxel has the same initial CO_2_ molar fraction, χ_c_, the value of *Δf*(χ_c_, *T*) is the same in both cases (as an example, we assume that *Δf*(χ_c_, *T*) > 0). However, Φ(ϕ, λ) is different in both cases because case (1) leads to a local interface with positive curvature, whereas case (2) leads to a flat interface. Φ(ϕ, λ) is calculated with eqs 3 and 4 using the Moore neighborhood represented in blue dotted lines. It is straightforward to verify that the Glauber transition probability for solidification (*W*_h_) of case (1) is smaller than the one for case (2). If *Δf*(χ_c_, *T*) < 0, in order to ensure a similar outcome, Φ(ϕ, λ) = 1 + *λκ* has to be replaced by Φ(ϕ, λ) = 1 – *λκ*.

Overall, *ΔF* (as it appears in the Glauber transition probabilities) considers the tendency to favor one phase or another based on bulk free energy minimization and the tendency to reduce the surface energy by reducing surface curvature (or surface area). In Figure 4, we present a schematic example in which, although the two liquid-to-solid transitions have an equal bulk free energy change, their Glauber transition probabilities are different because one leads to a flat interface and the other to a less favorable curved one. When λ = 0 (i.e., surface tension is ignored), Φ(ϕ, λ = 0) = 1, and the change in bulk free energy is the phase transformation determining factor. Thus, λ is a free parameter that is changed to explore the dynamics of this model.

#### Mass Transport

The mass transport mechanism considered in this work is diffusion. However, because the rate of CO_2_ diffusion through the hydrate-water interface decreases rapidly relative to the diffusion in the liquid water region, and it is several orders of magnitude smaller in the solid hydrate structure,^78^ mass transport via diffusion is considered only between voxels belonging to the water region. In other words, diffusion phenomena along the interfacial and solid hydrate regions and between such two regions are neglected.

**Figure 5:**
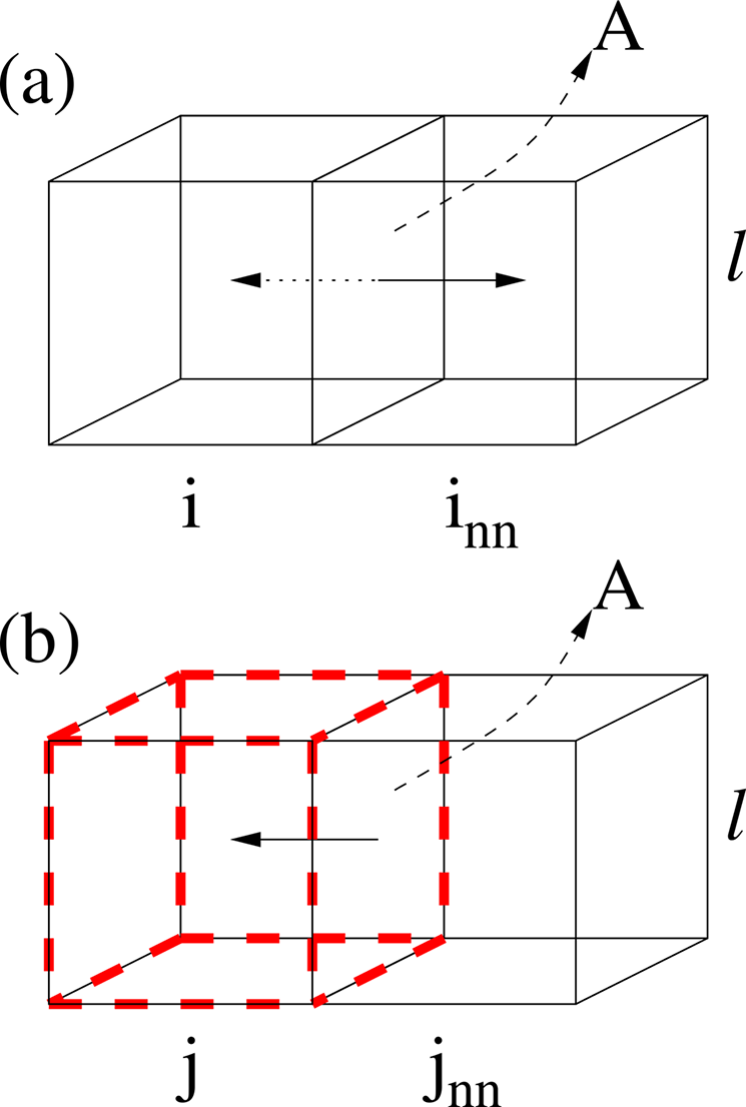
Schematic of the diffusion process for CO_2_ dissolved in voxels filled of water. Both voxels have the same constant volume *V* = *l*^3^, and the flux is through the area *A* = *l*^2^ connecting both voxels. The side length of the voxels is given by *l*, where, as mentioned before, *l* is the interface thickness. The direction of the flux depends on the concentration difference between voxels and is accepted, provided the system’s total energy is minimized. (b) Schematic of the interfacial attachment process (i.e., uptake of CO_2_ at the interface). This is similar to (a) but with *j* being an interfacial voxel (represented by the red dashed line) with a neighbor *j*_nn_ in the liquid phase. In this case, the net CO_2_ flux is only in one direction, namely, from voxel *j*_nn_ in the water region to the interfacial voxel, *j*.

Modeling diffusion is achieved using a probabilistic implementation of Fick’s first law.^61,62^ We assume that voxel-to-voxel diffusion occurs only between the first nearest neighbors voxels (von Neumann neighborhood). The voxels, however, can differ in composition. Thus, at each time step, *Δt*, one of the first nearest neighbors, *i*_nn_, is chosen randomly for each voxel, *i*, where *i* runs from 1 to the total number of water voxels in the system. Then, the net flux of CO_2_ molecules from voxel *i* to voxel *i*_nn_ is given as
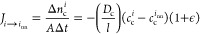
5provided *c*_c_^*i*^ > *c*_c_^*i*_nn_^. In this equation, *c*_c_^*i*^ = *n*_c_^*i*^/*V* and *c*_c_^*i*_nn_^ = *n*_c_^*i*_nn_^/*V* are the CO_2_ concentrations in the voxels expressed in terms of the corresponding numbers of moles *n*_c_^*i*^ and *n*_c_^*i*_nn_^ and the voxel volume *V* = *l*^3^. The term *A* = *l*^2^ is the area through the flux of CO_2_ molecules pass during a time *Δt*, *D*_c_ is the diffusion coefficient of CO_2_ in water, *l* is the side length of the voxels, and *Δn*_c_^*i*^ = *n*_c_^*i*^(*t* + *Δt*) – *n*_c_^*i*^(*t*). ϵ represents a random number from a Gaussian distribution with mean equal zero and standard deviation σ ≪ 1. If instead, *c*_c_^*i*^ < *c*^*i*_nn_^, the flux is performed from voxel *i*_nn_ to voxel *i* according to
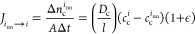
6where *Δn*_c_^*i*_nn_^ = *n*_c_^*i*_nn_^(*t* + *Δt*) – *n*_*c*_^*i*_*nn*_^(*t*). In this work, we relate the moles to the molar fraction as  and , with *v*_m_ being an average molar volume of water with dissolved CO_2_. In order to keep the volume of each voxel constant the CO_2_ flux is compensated by a water flux in the opposite direction. Finally, either of the two fluxes is accepted with probability
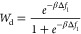
7where Δ*f*_l_, which is obtained from the free energy density of the liquid water with dissolved CO_2_, *f*_l_ (see Appendix), is the change of free energy of the system due to the diffusion process and β = (*k*_B_*T*)^−1^. Figure 5a presents a schematic example of two voxels connected by a common area *A* through which the flux of CO_2_ molecules and water occurs. The compositions of the voxels involved in the diffusion process are updated according to eqs 5 or 6, ensuring that χ_c_ + χ_w_ = 1 in each voxel.

#### Interfacial Attachment Kinetics

We assume a concentration-dependent driving force for the CO_2_ uptake (i.e., adsorption) at the solid hydrate-liquid water interface (i.e., integration layer).^23,24,79−88^ Because the hydrate film is present at the start of the simulations, this driving force is assumed to be the difference between CO_2_ concentration in liquid water near the interface and the CO_2_ concentration in bulk liquid water. Thus, we consider a net molar flux of CO_2_ molecules toward an interfacial voxel *j* as given by
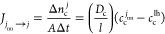
8where *n*_c_^*j*^ is the number of CO_2_ moles in voxel *j*. As before, *A* = *l*^2^ is the area through which the flux of CO_2_ molecules pass during a small time interval *Δt*, *D*_c_ is the diffusion coefficient of CO_2_ in water, and *l* is the side length of the voxels. *c*_c_^*j*_nn_^ = *n*_c_^*j*_nn_^/*V* denotes the CO_2_ concentration of voxel *j*_nn_ in the water region, with that voxel being a first nearest neighbor of interfacial voxel *j* (von Neumann neighborhood). Note that *j* runs from 1 to the total number of interfacial voxels. *c*_c_^lh^ = *n*_c_^lh^/*V* is the concentration of CO_2_ in liquid water near the interface that is in equilibrium with CO_2_ hydrates as expressed in terms of the voxel volume and number of moles, . In this work, the molar fraction χ_c_^lh^ will be assumed to be given by the liquidus line of the equilibrium phase diagram presented in the Appendix. The implementation of eq 8 is justified from the steady state mass balance resulting from the equality between the net integration (or so-called enclathration) rate of CO_2_ molecules into the partially formed cages at the interface, and the net molar flux toward that interface.^51,52,87^

For all *ΔT* values explored in this work, *c*_c_^*j*_nn_^ ≥ *c*_c_^lh^, and the net flux of CO_2_ molecule occurs from voxel *j*_nn_ to the interfacial voxel *j* (see Figure 5b). In our simulations, the maximum value that *c*_c_^*j*_nn_^ can have is that of the CO_2_-in-water solubility. Note also that Δ*n*_c_^*j*^ = *n*_c_^*j*^(*t* + *Δt*) – *n*_c_^*j*^(*t*), , and . Because the volume is conserved, the CO_2_ flux is compensated by a water flux in the opposite direction. The compositions of the voxels involved in the attachment process are updated according to eq 8, ensuring that χ_c_ + χ_w_ = 1 in each voxel.

## Numerical Implementation

**Figure 6:**
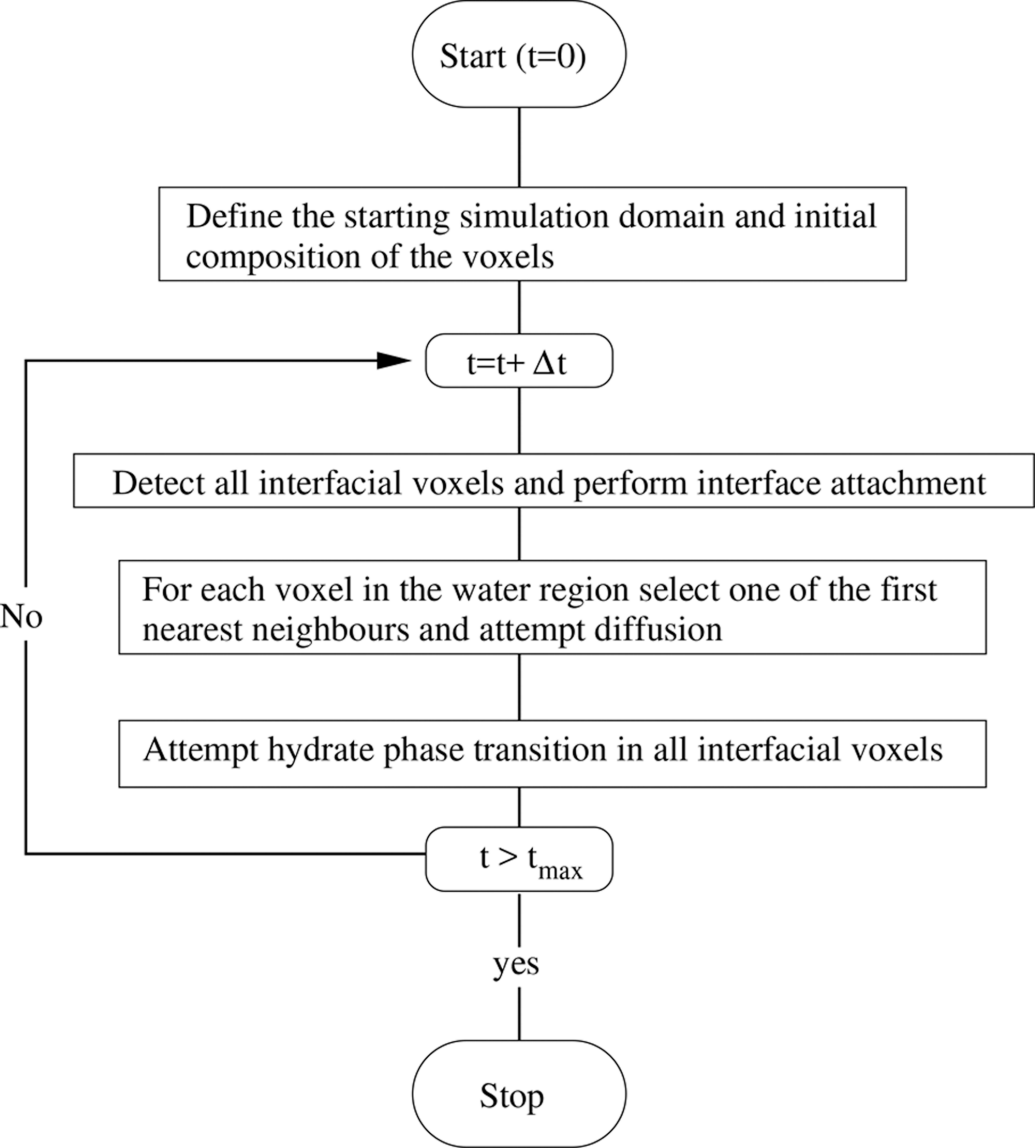
Flowchart for the main steps involved in the hybrid probabilistic CA simulation. At each step, *Δt*, the attachment, diffusion, and phase transformation processes proceed sequentially.

The dimensionless parameter,
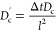
9from eqs 5, 6, and 8 defines the length and time scales of the system. We will use *D*_c_ = 10^–9^ m^2^/s.^62,89^ By choosing the iteration time as *Δt* = 1/9 × 10^–9^ s and the side length of the voxels as *l* = 10^–9^ m, the dimensionless diffusion coefficient is fixed at *D*_c_^′^ = 1/9.^61,62^ The simulation starts at *t* = 0 s with a given domain configuration and composition of the associated voxels. Then within each iteration time *Δt*, the phase transformation, diffusion, and attachment processes are implemented to update the state and composition of the voxels. A flowchart of the steps involved in the simulation is shown in Figure 6.

A simulation domain of total volume *V* = *Nl*^3^ is defined, where *N* is the total number of voxels in the domain. As presented in Figure 3, these voxels have an equal volume of *V* = *l*^3^ which remains constant during our simulations. Initially, the whole domain is divided into three regions. The first one represents the CO_2_ hydrate thin film and has a volume of *V*_h_ = *N*_h_*l*^3^, where *N*_h_ is the number of solid hydrate voxels. The second region corresponds to the water with dissolved CO_2_ voxels having at least one nearest neighbor solid hydrate voxel (i.e., hydrate-water interface) and has a total volume of *V*_in_ = *N*_in_*l*^3^, where *N*_in_ is the total number of interfacial voxels. The third region is the one containing the rest of the water with dissolved CO_2_ voxels and has a total volume of *V*_w_ = *N*_w_*l*^3^, in such a way that *V* = *V*_h_ + *V*_in_ + *V*_w_ = (*N*_h_ + *N*_in_ + *N*_*w*_) *l*^3^ = *Nl*^3^. Although *N* is constant, *N*_h_, *N*_in_, and *N*_w_ evolve in time due to the hydrate phase transformation of the interfacial voxels. However, initially we assume that *N*_in_ < *N*_h_ < *N*_w_ (note that in Figure 3, *N*_in_ = *N*_h_ < *N*_w_).

After setting up the simulation domain and initializing the composition of the voxels, time is advanced by *t* + *Δt*. Then, the interfacial voxels are identified, and the interfacial attachment process is performed for each interfacial voxel. The mass transport between the water region voxels proceeds by selecting one first nearest neighbor for each voxel and realizing the diffusion of CO_2_ and water molecules. The current simulation step ends with the solidification attempt of all interfacial voxels. After that, the time step increases again by *Δt*. As specified in Figure 6, the procedure is repeated until a final stipulated time is reached.

## Simulation Results

The hybrid probabilistic CA modeling approach is developed to model the crystal growth morphology of gas hydrate during its formation in stagnant water with a dissolved gas hydrate-forming substance. As a case study, we consider the variations in the morphology of sI CO_2_ hydrate. The primary intent is to correlate those variations with the degree of subcooling, *ΔT*, and the local interface curvature, quantified by the dimensionless parameter λ and the variable term κ.

The simulations start from a preformed CO_2_ hydrate thin film exposed to water with dissolved CO_2_. We will assume that for all *ΔT* values explored in this work, the initial CO_2_ molar fraction in all voxels of the interface and water regions is around the constant value χ_c_^o^ > χ_c_^lh^, where, χ_c_^lh^ will be a decreasing function of *ΔT* (see the liquidus line of the isobaric phase diagram in the appendix section). In the following, all the results will be expressed in molar fractions, and in all figures the time will be presented in units of microseconds (μs). Following Tegze et al.,^56^ we will perform simulations for conditions typical for seabed reservoirs. Thus, the pressure will be *P* = 6.2 MPa, and the triple equilibrium temperature will be assumed to be *T*_eq_ = 282.5 K (see Figure 15 in the appendix section).

With the length and time scales of the model defined implicitly by the parameter *D*_c_^′^, the only parameters left for varying are the subcooling, *ΔT*, and the strength of the local curvature effect, λ. Increasing *ΔT* leads to an increment of the CO_2_ uptake at the interface and the subsequent hydrate growth rate, while λ tends to reduce the surface area of the interface by forcing it to take a flat shape. In our simulations, each voxel of the water and interfacial regions are randomly initialized with a molar fraction of χ_c_^o^ = 0.029 + δ, where δ is a random number from a Gaussian distribution with mean equal zero and standard deviation σ = 10^–4^.^61,62^ This initial molar fraction represents the amount CO_2_ in water in equilibrium with the vapor or gas phase (or solubility of CO_2_ in water). As Figure 17 in the Appendix shows, this quantity impacts the hydrate crystal formation, growth and morphology.^90,91^ In all cases, we consider an initial hydrate thin film with a thickness of 2 × 10^–3^ μm in the *x* direction, extended all along the *y* direction.

### Morphology

**Figure 7:**
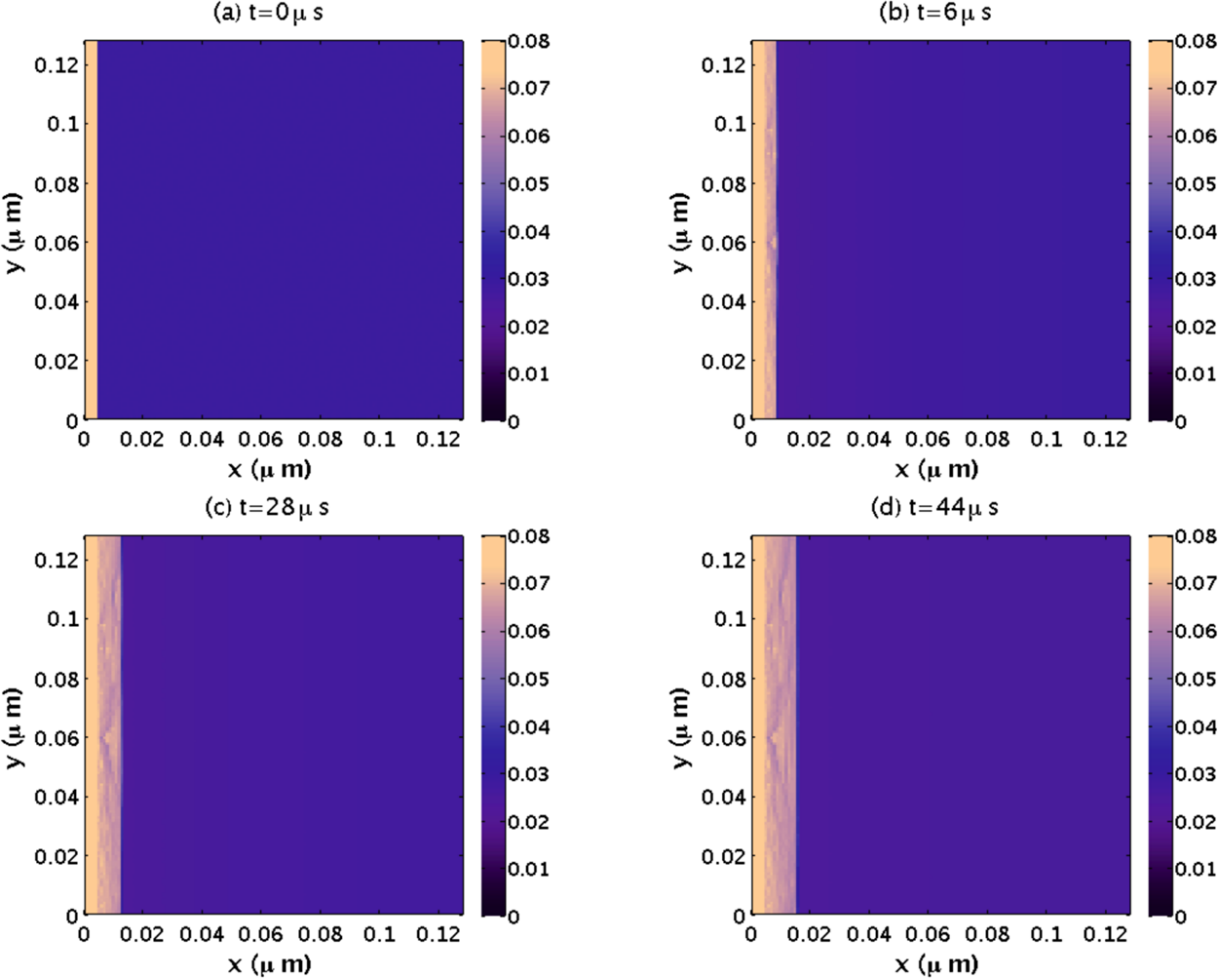
(a–d) Simulated sI CO_2_ hydrate planar front growing from a seeded hydrate thin film toward liquid water with dissolved CO_2_, for *ΔT* = 1.1 K and λ = 0.5. Shown is the CO_2_ molar fraction in the hydrate and the liquid water. In panel (a), the molar fraction in the initial hydrate thin film is in white and the rest of the domain corresponds to liquid water’s CO_2_ molar fraction. In this case, *L*_*x*_ = *L*_*y*_ = 0.128 μm. Other parameters are as indicated in the main text.

**Figure 8:**
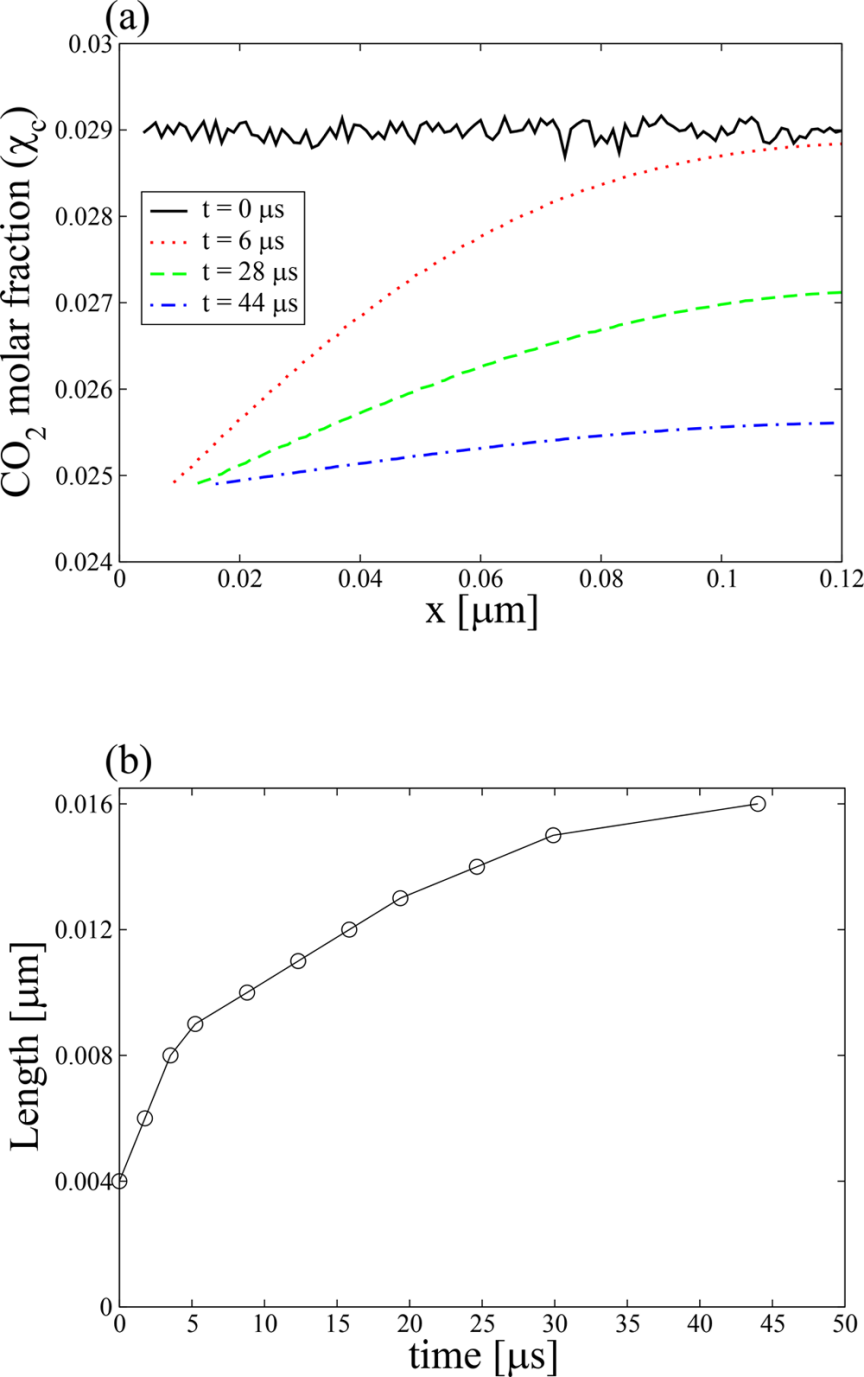
(a) The CO_2_ molar fraction profile in the water region at *y* = 0.064 μm and along the *x* direction for the panels presented in Figure 7. The black solid line corresponds to *t* = 0 μs, the red dotted line to *t* = 6 μs, the green dashed line to *t* = 28 μs, and the blue dash-dotted line to *t* = 44 μs. The CO_2_ molar fraction minimum in water moves to the right because the hydrate front moves in that direction. (b) Position of the planar front of Figure 7 as a function of time for parameter values implemented in panel (a).

In Figure 7, we present the spatiotemporal evolution of a CO_2_ hydrate planar front, for *ΔT* = 1.1 K and λ = 0.5. Although not shown in the figure, the planar front moves slowly beyond 44 μs but eventually stops when there is no driving force to continue growing. To better appreciate the mechanism behind the CO_2_ hydrate planar front growing presented in Figure 7, we plot in Figure 8a the CO_2_ molar fraction profile in the water region at *y* = 0.64 μm and along the *x* axes, for the four panels of Figure 7. As the figure shows, at *t* = 6 μs (red dotted line), the CO_2_ molar fraction in the liquid water equals the initial average value of χ_c_^o^ = 0.029, at the far right; the CO_2_ molecules have not yet diffused from those regions toward the interface. Near the interface, a minimum rapidly evolves. Thus, the planar front motion is viewed as a moving interface determined by the diffusion of CO_2_ molecules toward that interface from the bulk liquid phase. This diffusion is driven by the difference in CO_2_ molar fractions between the bulk and the region near the interface. As the diffusion continues, the CO_2_ molar fraction at the far right decreases, and the growth eventually stops once the concentration gradient is zero. In Figure 8b, the position of the front is tracked by following the minimum CO_2_ molar fraction value near the interface. The position of interface follows a power law (length ∝ *t*^1/2^), indicating a planar crystal diffusion-controlled growth in which the planar crystals do not grow steadily. Thus, its growing velocity decreases proportionately to *t*^–1/2^.^72^

**Figure 9:**
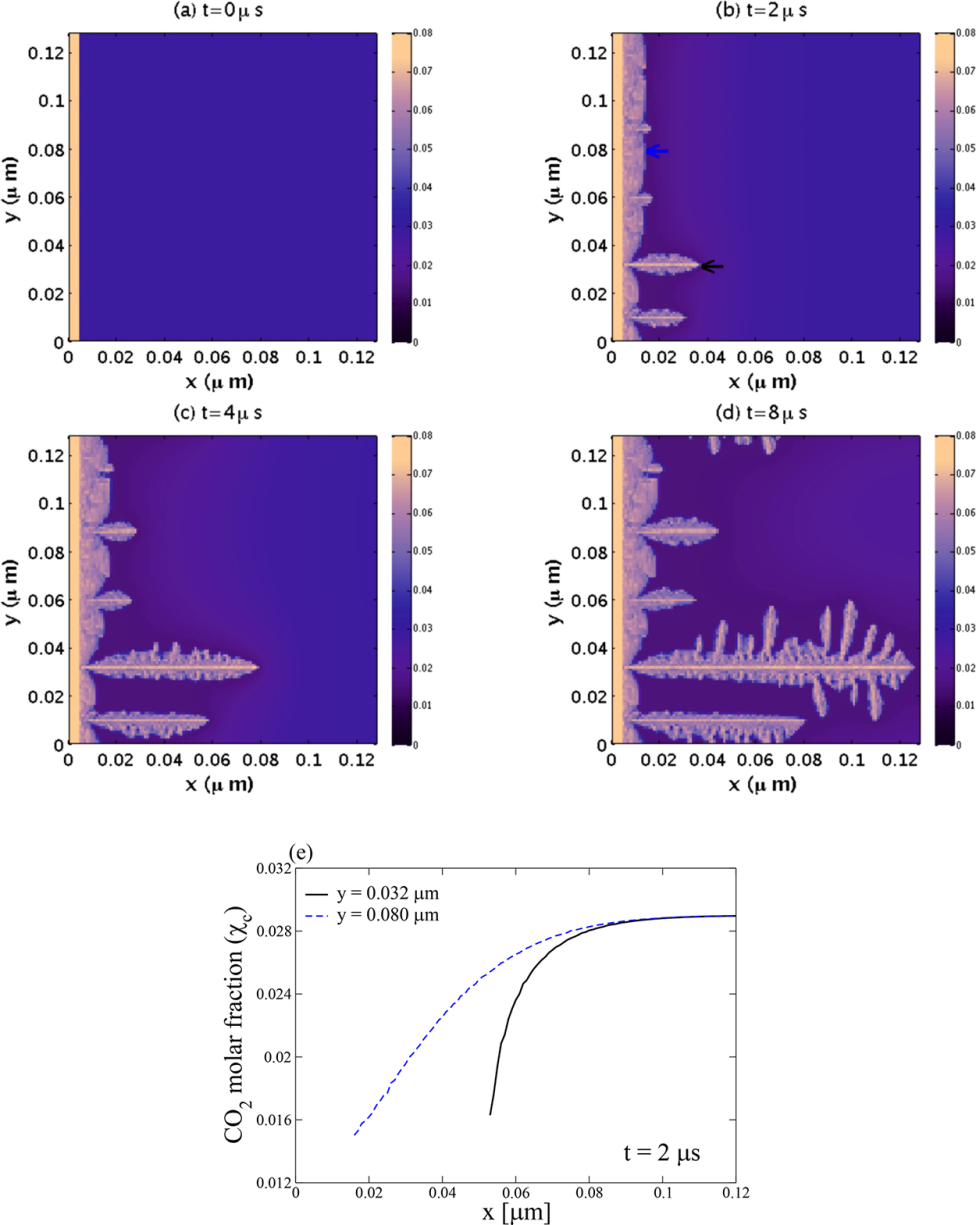
Panels (a), (b), (c), and (d) show the temporal evolution of simulated sI CO_2_ hydrate growth from a seeded hydrate thin film toward liquid water with dissolved CO_2_, for *ΔT* = 9.1 K and λ = 0.5. Panel (e) shows CO_2_ molar fraction profiles in the water region at *y* = 0.032 and 0.080 μm, along the *x* direction for parameter values of panel (b). The arrows in panel (b) indicate the position of the minima molar fractions in panel (e). In this case, *L*_*x*_ = *L*_*y*_ = 0.128 μm. Other parameters are in the main text.

In Figure 9, we present the spatiotemporal evolution of CO_2_ hydrate from the hydrate film, but now for *ΔT* = 9.1 K, keeping the same value for λ = 0.5. As the figure shows, initially, a planar front evolves toward the liquid water region. However, needle-like crystals eventually form due to local random fluctuations at the interface and advance deeper into the liquid water phase with a faster local growth rate than the neighboring planar regions. This behavior indicates that the planar front is unstable for such a large degree of subcooling.

While some crystals grow with a parabolic needle geometry, others develop side branches forming what are known as parabolic dendrites.^72^ As Figure 9e shows, the difference in growth rate between the planar regions and the growing crystals is because the CO_2_ molar fraction gradient is more prominent in the water region in front of the crystal tips. A stronger gradient implies a more significant flux of CO_2_ molecules toward the interface, resulting in a larger local hydrate phase transformation probability and hence a faster growth rate. This positive feedback mechanism is known as Mullins-Sekerka instability.^92^ It often leads to pattern formation, one of the most common ones being the dendrite shape pattern in which a tree-like structure develops. This instability also causes columnar, needle, and sword-like patterns observed during the growth of gas hydrate crystals.^23−25^

The simulations above suggest that large enough values of *ΔT* destabilize, through the Mullins-Sekerka mechanism, the sI CO_2_ hydrate planar fronts that move toward liquid water with dissolved CO_2_. On the other hand, large values of parameter λ are expected to act as a stabilizing factor for the planar interface because its goal is to reduce the total interfacial area to its minimum (i.e., the interfacial area of a planar front). Thus, to better quantify the interplay between these two competing model parameters, let us introduce the following quantity,
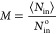
10where *N*_in_ is, as mentioned in previous sections, the total number of interfacial voxels at a given time step, and *N*_in_^o^ is a constant expressing the total number of interfacial voxels for the special case of a planar interface. ⟨*N*_in_⟩ is the average value of *N*_in_ over the number of time steps. Thus, for a growing planar interface, *M* = 1 (i.e., *N*_in_ = *N*_in_^o^), while for an irregular one, *M* > 1 (i.e., *N*_in_ > *N*_in_^o^).

**Figure 10:**
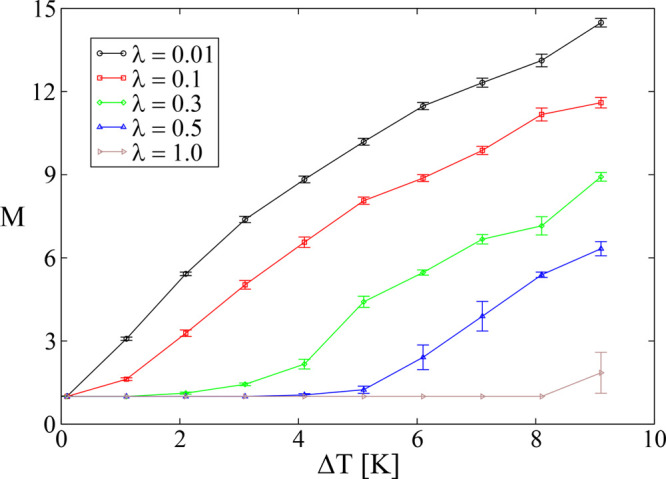
Quantity *M*, quantifying front morphology (see eq 10), as a function of the degree of subcooling *ΔT*, for several values of λ. Each simulation point is an average over four independent realizations, and the error bars denote ± one standard deviation around that average. The total simulation time for each data point is *t* = 24 μs. In this case, *L*_*x*_ = *L*_*y*_ = 0.128 μm. Other parameters are as indicated in the main text.

**Figure 11:**
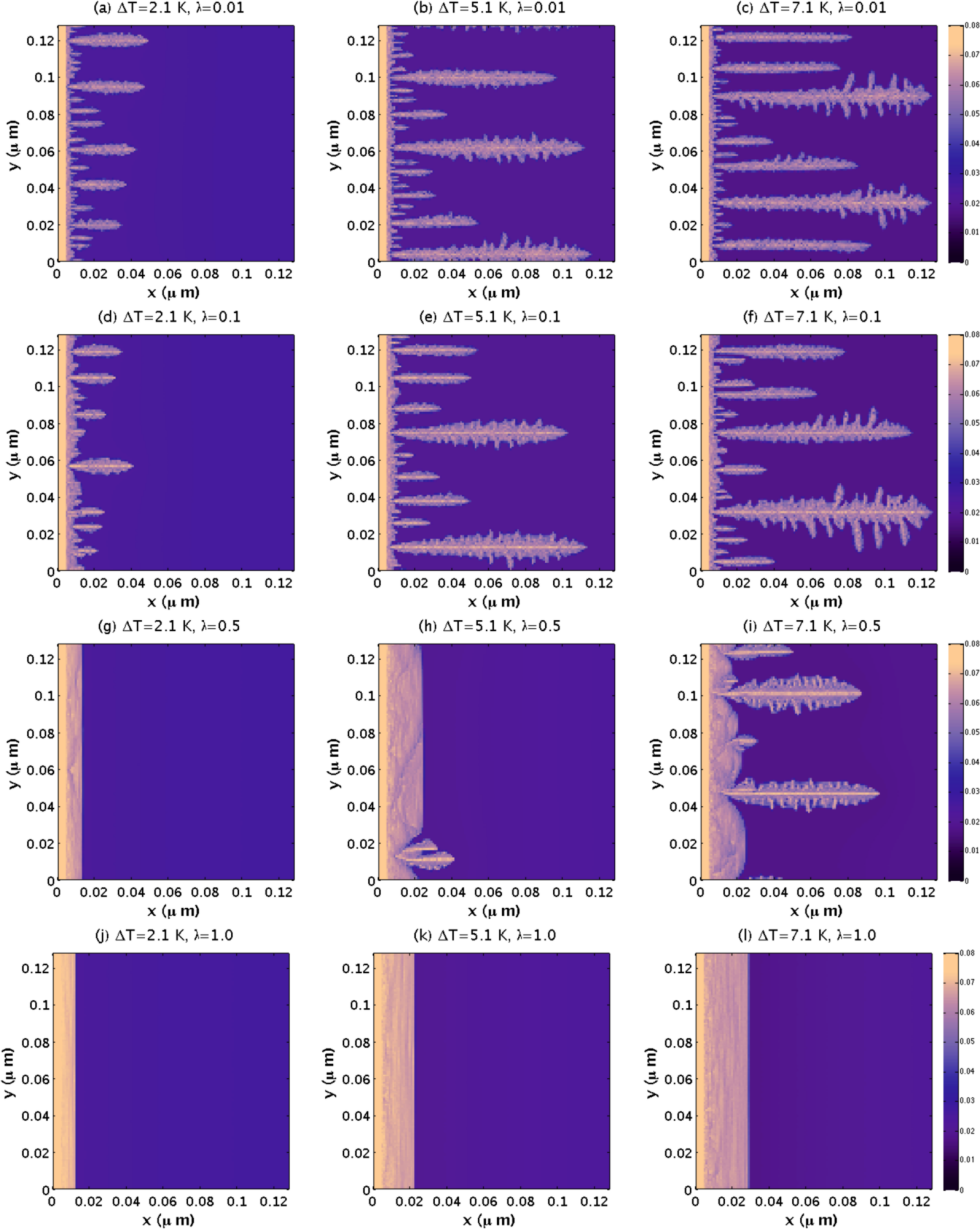
(a–l) Spatiotemporal evolution of the simulated sI CO_2_ hydrate growing from a seeded hydrate thin film toward liquid water with dissolved CO_2_, for several values of *ΔT* and λ. All parameters are as in Figure 10, and all panels are at *t* = 24 μs. After that time, the hydrate barely grew because the driving force for hydrate growth is minimal in some cases or even zero in others. The color bars of the most right panels apply to all panels.

From Figure 10, where *M* (see eq 10) is plotted as a function of *ΔT* for several λ values, it is evident that the extent of *ΔT* for which *R* is equal or close to the ideal value of unity increases with λ. This behavior indicates that, indeed, the stability of the planar front increases with λ and decreases with *ΔT*. In Figure 11, we present the spatiotemporal evolution of sI CO_2_ hydrate growth for some of the data points of Figure 10. For λ = 0.01, the planar front is unstable for all simulated values of *ΔT*. In particular, for *ΔT* = 2.1 K, parabolic needle-like crystals emerge from the initial hydrate thin film. For *ΔT* = 5.1 K, the needle-like crystals penetrate deeply into the water region and become parabolic dendrites by developing side branches. Finally, for *ΔT* = 7.1 K, the side branches of the parabolic dendrites grow larger, and the crystals penetrate further into the water region. The figure also shows that the number of crystals that grow into the water region increases with *ΔT* for all values of λ except for λ = 1, where only planar fronts are observed. The transition from irregular crystal growth to planar crystal growth as a function of λ, for a given *ΔT*, occurs gradually.

### Hydrate Conversion

**Figure 12:**
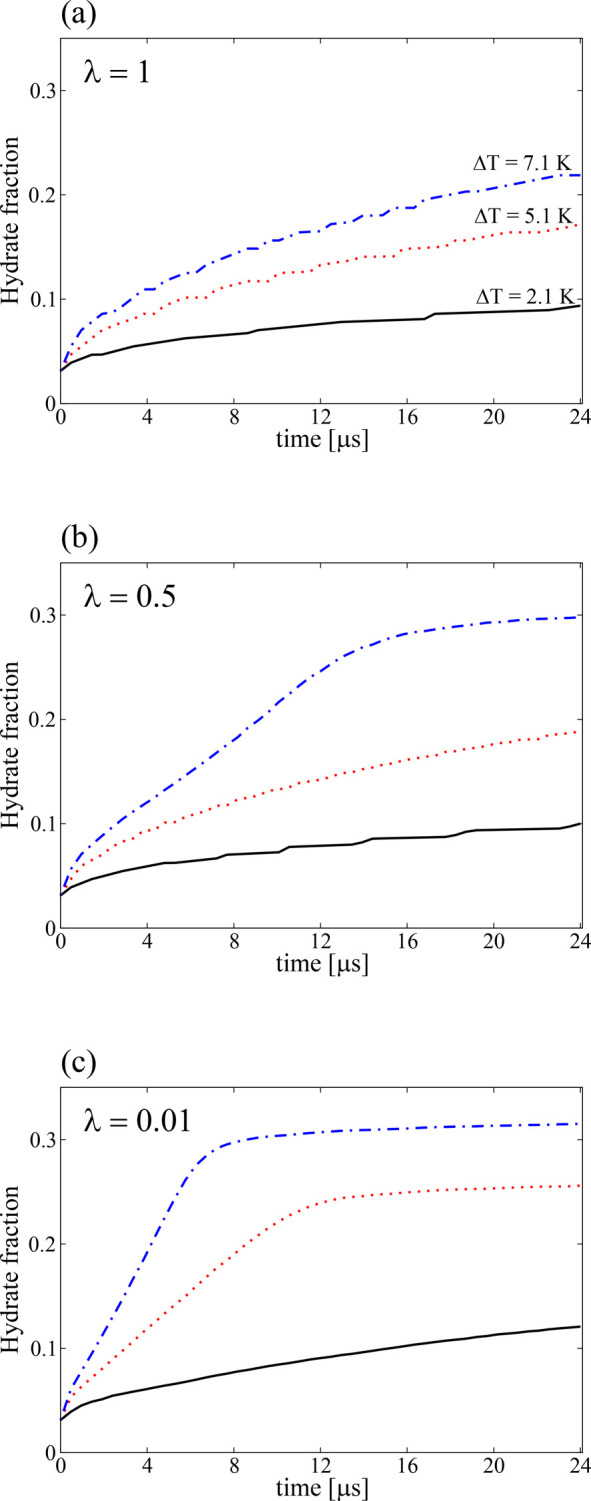
(a–c) Hydrate fraction as a function of time, for parameter values of Figure 11. This fraction is calculated as the number of voxels in the solid phase (hydrate) divided by the total number of voxels. The black solid lines are for *ΔT* = 2.1 K, the red dotted lines are for *ΔT* = 5.1 K, and the blue dash-dotted lines are for *ΔT* = 7.1 K.

We now explore how the fraction of hydrate within the simulation domain evolves in time. This quantity is obtained by counting the number of solid hydrate voxels at each time step, including the initial hydrate thin film voxels, and dividing it by the total number of voxels. Thus, a hydrate fraction of 1 means the whole simulation domain is CO_2_ hydrate. Figure 12 presents this conversion rate as a function of time for three representative values of λ and *ΔT*. For λ = 1 (see Figure 12a), the hydrate grows as a planar front for all the chosen *ΔT* values (see bottom panels in Figure 11), and hydrate fraction is in proportion to *t*^1/2^. In other words, we get a planar crystal diffusion-controlled growth.^72^ For λ = 0.5, with *ΔT* = 2.1 and 5.1 K (see Figure 12b), the hydrate fraction also exhibits a planar crystal diffusion-controlled growth, as Figure 11g and Figure 11h show. Although in Figure 11h a protrusion emerges at the end of the simulation, the hydrate grows as a planar front during most of the simulation.

For *ΔT* = 7.1 K, the dynamic evolution of the hydrate fraction exhibits a different behavior. Early on diffusion-controlled planar crystal growth is dominant. However, after a certain simulation time, the hydrate fraction suddenly increases steadily (in proportion to *t*). This behavior is typical of diffusion-controlled growth of needle crystals with parabolic tips, where the growth occurs mainly at the tip region.^72^ Eventually, the hydrate fraction reaches a plateau because the driving force to growth diminishes. For λ = 0.01 and *ΔT* = 7.1 K (see Figure 12c), the hydrate fraction increases steadily with time until a plateau starts to emerge. As Figure 11c shows, needle-like and dendrite parabolic crystals form for such values of subcooling. The hydrate fractions for *ΔT* = 5.1 and 2.1 K also increase steadily with time after an initial short time, during which the hydrate fraction evolution resembles the one of a planar front. It is interesting to point out that the curve for *ΔT* = 5.1 K reaches a plateau, while the one for *ΔT* = 2.1 K does not. This behavior occurs because the one with *ΔT* = 5.1 approaches a zero driving force within the time scales considered, whereas the other still does not. Panels (a) and (b) in Figure 11 show how the crystals grow for the two subcooling values just mentioned. As expected, the parabolic crystals for *ΔT* = 2.1 K grow more slowly because of a smaller associated driving force.

### Larger Simulation Domain

**Figure 13:**
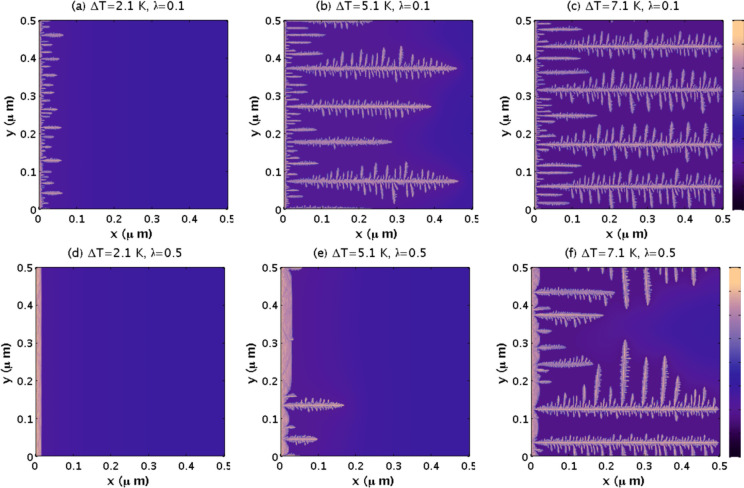
(a–f) As in Figure 12, but for larger scale, *L*_*x*_ = *L*_*y*_ = 0.5 μm. All panels are at *t* = 44 μs. The color bars of the most right panels apply to all other panels.

**Figure 15:**
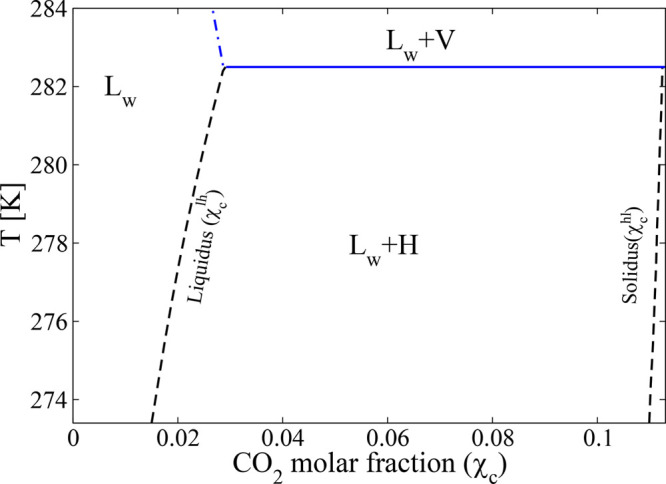
Equilibrium phase diagram for the liquid water-CO_2_ system at a pressure of *P* = 6.2 MPa. In the region denoted by L_w_, liquid water with dissolved CO_2_ is the stable phase. Liquid water with dissolved CO_2_ coexist with vapor CO_2_ in the region, L_w_ + V, whereas in the region denoted by L_w_ + H, water with dissolved CO_2_ can coexist with a sI CO_2_ hydrate with large cages fully occupied and small ones empty. The two dashed lines are obtained from the common-tangent method using the free energy densities as presented in Figure 14 and outlined in this Appendix. The liquidus line (χ_c_^lh^) is the CO_2_ molar fraction in water in equilibrium with CO_2_ hydrate (left black dashed line). The solidus line (χ_c_^hl^) is the CO_2_ molar fraction in hydrate in equilibrium with the water with dissolved CO_2_ (right black dashed line). The assumed triple vapor-hydrate-water equilibrium temperature *T*_eq_ = 282.5 K is presented as a solid blue line, while the CO_2_-in-water solubility line is shown as a blue dash-dotted line. An exact calculation of the latter two lines requires a free energy expression for the vapor CO_2_. Although not shown in this figure, after the solidus line, there is a small region where only solid hydrate, H, is stable. After that region, a coexisting region between hydrate and vapor CO_2_ exists (H + V). For details on this phase diagram, we refer to refs (54−57 and 76).

**Figure 17:**
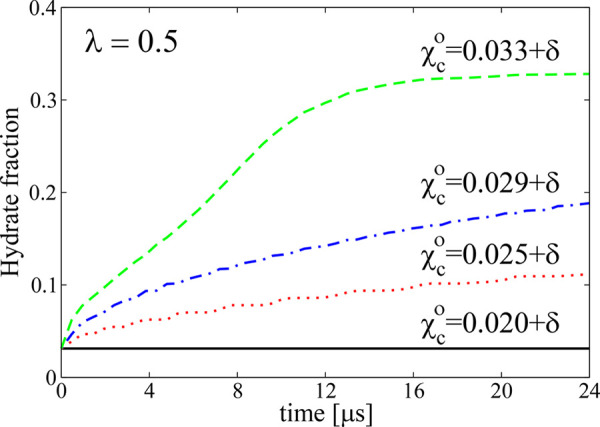
Hydrate fraction as a function of time for several initial molar fractions of CO_2_ in the water domain. The hydrate fraction is calculated as the number of voxels in the solid phase (hydrate) divided by the total number of voxels in the simulation domain. Thus, a hydrate fraction equal to 1 means the whole simulation domain is CO_2_ hydrate. These simulations are for *ΔT* = 5.1 K, and, as mentioned in the main text, λ, is a dimensionless parameter quantifying the impact of the curvature of the hydrate-water interphase. Each voxel of the water simulation domain is started with a CO_2_ molar fraction given by χ_c_^o^ = χ + δ, where δ is a random number from a Gaussian distribution with a mean equal zero and standard deviation σ = 10^–4^ and χ = 0.020, 0.025, 0.029, and 0.033. The corresponding molar fraction of water is χ_w_^o^ = 1 – χ_c_^o^.

Finally, in Figure 13, we plot the spatiotemporal evolution of sI CO_2_ hydrate toward liquid water for a larger simulation domain. As in previous cases, the stability of the planar front is determined by the competition between the Mullins-Sekerka destabilization mechanism for large *ΔT* and the surface tension stabilization factor quantified by the parameter λ. However, in this case, the number of available CO_2_ molecules in the water domain is higher, and the hydrate growth driving force lasts longer. Thus, the model yields larger parabolic dendrites, with side branches that develop even smaller branches, separated by barely developed needle-like parabolic crystals. The density of crystals increases with *ΔT* and decreases with λ.

## Discussion and Conclusions

CO_2_ hydrate technologies offer potential alternative solutions to energy and climate related challenges.^69^ However, a clear understanding and prediction of morphological variations of CO_2_ hydrate crystals are necessary to support the further development of such technologies. Hydrate crystal shape should strongly influence the kinetics of the hydrate formation process, and it should be taken into account in designing equipment, such as transportation devices and reactors.

In this work, we implemented a hybrid probabilistic CA modeling framework to explore morphology variations of CO_2_ hydrates growing in stagnant liquid water presaturated with CO_2_. The framework offers an alternative to the PF modeling approach for gas hydrate growth in terms of implementation simplicity and computational efficiency.^54−57,76^ These two aspects are essential when modeling gas hydrate formation in multiphase flow systems where modeling frameworks of gas hydrate growth kinetics must be coupled to computational flow modeling tools.^67^ The modeling approach developed and implemented in this work constitutes a first step toward an accurate, efficient, and easy-to-implement computational framework for investigating gas hydrate crystal morphology.

Our simulations focused on the sI CO_2_ hydrate crystal growth and morphology variations toward aqueous CO_2_ solution from a preformed sI CO_2_ hydrate thin film and under conditions typical to oceanic natural gas hydrate reservoirs. Under these conditions, the natural gas hydrates can be converted into the significantly more stable CO_2_ hydrate in the presence of liquid CO_2_ or aqueous CO_2_ solution while natural gas is released.^14,56^ Phase transformation and attachment kinetics at the hydrate growing interface and mass transport by diffusion in liquid water are the main physical processes accounted for in our modeling approach. The framework is developed based on the assumption that gas hydrate morphology is controlled by the mass transfer of the CO_2_ molecules dissolved in the bulk of the liquid water toward the growing hydrate crystal surfaces, and that the corresponding rate of mass transport increases with the level of subcooling, *ΔT*.^23−25^ This mass transport results from the difference in CO_2_ concentration between the bulk of the aqueous solution and that near the hydrate-water interface.^23−25^

We explored the morphological growth changes as a function of Δ*T* and properties of the hydrate-water interface, such as surface tension and curvature. The modeling approach allowed us to explore the stability of the moving sI CO_2_ hydrate planar fronts as a function of *ΔT* and parameter λ, which quantifies, in a qualitative manner, the impact of surface tension and curvature. A higher value of parameter λ acts as a stabilizing factor of the flat interface, while a higher *ΔT* tends to destabilize it. We found that the planar interfaces follow a power law, length ∝ *t*^1/2^. We also found that the planar fronts become destabilized for a particular combination of *ΔT* and λ, giving rise to parabolic needle-like and parabolic dendrite crystals. As expected for parabolic crystals, the crystal growth proceeds steadily with time (i.e., in proportion to *t*).^72^ In many applications, a planar front could be more desired, and in this respect, thorough experimental and computational investigations of the impact of *ΔT* and surface tension on the stability of gas hydrate planar fronts could be relevant.

An appealing feature of our CA model is that it reproduces complex hydrate morphologies under diffusion-controlled growth. It is possible to extend the framework to study the problem of CO_2_ hydrate melting. Although diffusion across and along the interface is neglected, the model can be readily incorporated into the framework. A further extension to a whole 3D simulation domain is possible, and the domain boundary conditions can be easily adapted to the problem under consideration. Other developments of our CA modeling framework could also incorporate the impact of the temperature rise at the interface due to phase transformation and its removal by thermal diffusion.^61,62^ For a fixed temperature, the impact of increasing pressure on the morphology variation can also be studied within this framework.^24^ Furthermore, the extension of the framework to study gas hydrate morphological variations in oil–water systems instead of the liquid CO_2_–water one studied in this work is an exciting avenue for future research as it is considering other hydrate formers.^93,94^ Applying our simulation approach to the case of CH_4_ hydrate crystal growth and morphology is a topic for future studies. In this case, bulk-free energy densities entering the transition probabilities of the hydrate phase transformation must be considered for solid CH_4_ hydrate and water with dissolved CH_4_. Another aspect to consider is that, given the differences in solubility of CO_2_ and CH_4_ in water, the kinetic gas hydrate growth in these two cases will differ.^55,95^ The solubility of CO_2_ in water is higher than the one of CH_4_;^55,95^ therefore, the kinetic rates of CO_2_ hydrate growth should be more prominent than the one of CH_4_ hydrate under the same operating conditions.^70,95^ A first step in comparing these two important hydrate formers has already been performed by Kvamme et al. by using the computationally costly PF approach combined with MD.^54^ One can also apply our computationally efficient framework to Xe, which has emerged as attractive laboratory alternative for studying hydrate formation and dissociation.^58,59^

Although this work’s hybrid probabilistic CA approach reproduces features of diffusion controlled gas hydrate growth, it uses lumped parameters to describe the impact of complex phenomena on that growth.^54−57,61,62,76^ It is however possible to derive the parameter λ governing the strength of the curvature effect on solidification and the noise terms ϵ added to the diffusion fluxes from first-principles.^61,62^ Another point that needs attention is a more rigorous treatment of the phase transformation along the water-hydrate interface concerning the time scale of solidification and hydrate stability in terms of cages occupancy.^61,62^ Addressing these issues will make the model more predictive. However, as mentioned above and already noted by Buanes et al.,^61,62^ the computational efficiency of the easy-to-implement CA modeling approach makes it a valuable supplemental tool to more rigorous approaches. Thus, for instance, PF theory could be used to calibrate the free parameters of the hybrid probabilistic CA model by doing simulations in small systems. Then the CA model can be used to perform simulations of larger systems in scales relevant to real experiments, which are not currently attainable using the PF framework.

Although CO_2_ hydrate is at the center point in hydrate technologies of CO_2_ capture and separation, the number of experimental studies on the crystal growth and morphology of CO_2_ is relatively scarce. We still can, however, compare our simulation results to published literature.^5,20,21,24,31−35,96,97^ For example, our simulations agree with the observation of CO_2_ hydrate growth and dendrite formation as reported by Tohidi et al. for pressures to 6.5 MPa.^35^ Our results are also consistent with the experimental observation of the transition from skeletal polyhedral or columnar to dendritic on CO_2_ hydrate crystals in liquid water in contact with gaseous CO_2_, as a function of the subcooling parameter.^5,23,24^ However, in contrast to the water–gaseous CO_2_ system, under our simulation conditions in which CO_2_ is expected to be in the liquid state, the driving force for CO_2_ hydrate formation may be higher,^32^ meaning that a low degree of subcooling will be needed to destabilize the planar hydrate front and produce dendrite-like morphologies.

## Figures and Tables

**Figure 14:**
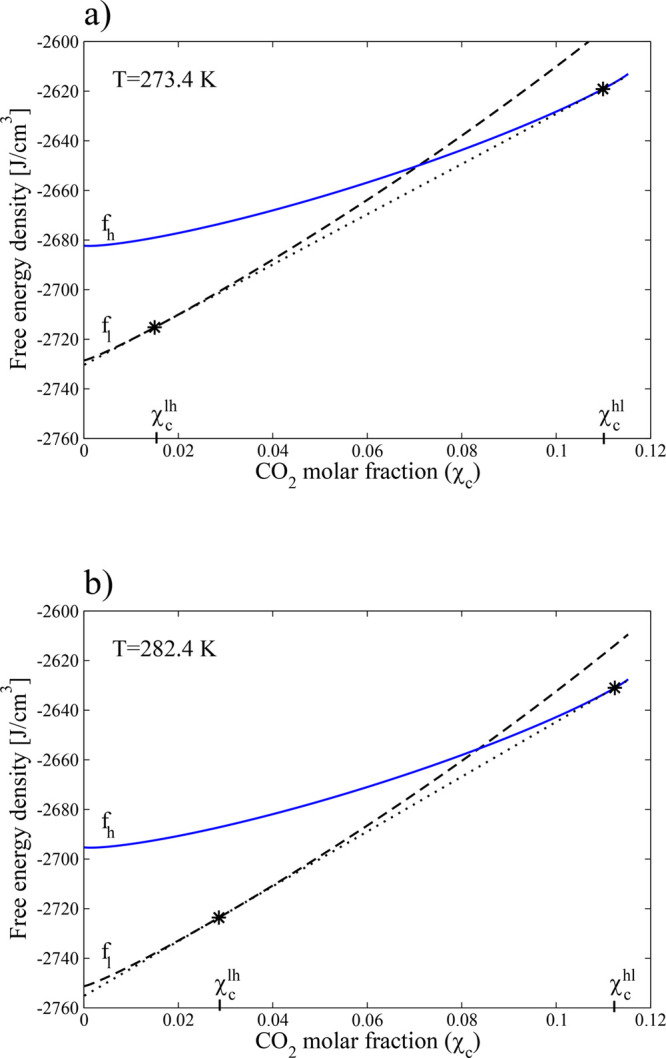
(a, b) Bulk free energy density as a function of the CO_2_ molar fraction for CO_2_ sI hydrate (solid blue lines, *f*_h_) and water with dissolved CO_2_ solution (dashed black lines, *f*_l_) as specified by eqs 11 and 18 free energy densities are for the case in which the CO_2_ molecules only occupy the large cages, with the small ones empty. The dotted line in both figures is the common tangent which expresses that the chemical potentials are equal in the two phases. The stars are the corresponding equilibrium CO_2_ compositions in the coexisting hydrate (χ_c_^hl^) and water (χ_c_^lh^) phases. χ_c_^hl^ is the CO_2_ molar fraction in the hydrate in equilibrium with water with dissolved CO_2_, while χ_c_^lh^ is the CO_2_ molar fraction in water in equilibrium with CO_2_ hydrate.

**Figure 16:**
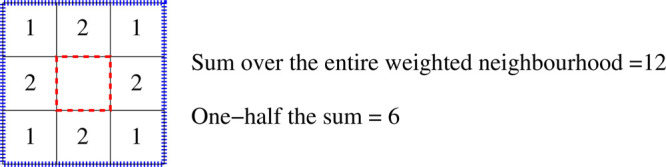
Template used in order to calculate the curvature of a flat surface. This curvature is taken to equal one-half the sum over the entire weighted neighborhood with all voxels in a solid hydrate state.

## Appendix

### Free Energy Densities

In this work, we adopt the free energy densities for liquid water with dissolved CO_2_ and CO_2_ hydrate implemented in refs (54−57, and 76). The free energy densities are parameterized by the CO_2_ molar fraction, χ_c_, the temperature, *T*, and the pressure, *P*. Thus, the free energy density of the liquid water with dissolved CO_2_ is given as

11

where *v*_m_ is the average molar volume (assumed independent of either phase or the concentrations), and *f*_l*,*w_ and *f*_l*,*c_ are the molar free energies of water and CO_2_ in the liquid solution, given as

12

and

13

where

14

is the molar free energy of pure water in kJ/mol, and

15

is the molar free energy of CO_2_ at an infinite dilution in kJ/mol. In previous equations,

16

and

17

*R* is the universal gas constant, *v*_w_ is the molar volume of water, *v*_c_ is the molar value of CO_2_, γ_l,w_(χ_c_) is the activity coefficient of water in the solution, γ_l,c_(χ_c_) activity coefficient of CO_2_ in the solution, and *P*_o_ is a reference pressure (normally 1 bar). Here, for simplicity we are assuming that *v*_c_ = *v*_w_ = *v*_m_ = 18 cm^3^/mol. The temperature *T* is in units of Kelvin. The coefficients are *l*_0_ = −109.0343, *l*_1_ = 37.7476 × 10^3^, *l*_2_ = −8.1591 × 10^6^, *l*_3_ = 63.0339 × 10^7^, *a*_1_ = −3.26384396, *a*_2_ = 1.662261661 × 10^2^, *a̅*_1_ = −2.52202459 × 10^4^, and *a̅*_2_ = 1.020418934 × 10^6^.^54−57,76^

Similarly, the free energy density of CO_2_ hydrate is given as

18

where the molar free energy of water in hydrate in kJ/mol is

19

and that of CO_2_ is,

20

where

21

is the occupancy, with ν_L_ = 3/23. In this case, a sI CO_2_ hydrate structure is assumed, with large cages fully occupied by CO_2_ molecules and small ones empty. In the previous expression,

22

is the molar free energy of the empty hydrate in kJ/mol, and

23

is the molar free energy of the CO_2_ inclusion.^54−57,76^ The coefficients are *s*_0_^0^ = −90.4838, *s*_1_^0^ = 27.4822 × 10^3^, *s*_2_^0^ = −6.1197 × 10^6^, *s*_3_^0^ = 47.8027 × 10^7^, *s*_0_^inc^ = −91.4060, *s*_1_^inc^ = 31.9655 × 10^3^, *s*_2_^inc^ = −6.4582 × 10^6^, and *s*_3_^inc^ = 47.8260 × 10^7^.^54,55,57,76,95^

### Common-Tangent Construction and Isobaric Phase Diagram

Given the functional forms of the free energies densities, *f*_l_ and *f*_h_, the equilibrium compositions, χ_c_^lh^ and χ_c_^hl^ are obtained from

24

and

25

eqs 24 and 25 express equality of the chemical potentials at equilibrium.

Figure 14 shows the free energy densities from eqs 11 and 18 for two different temperatures as a function of the CO_2_ molar fraction. Given these free energy densities, the CO_2_ equilibrium composition of the coexisting hydrate (χ_c_^hl^) and water (χ_c_^lh^) phases can be obtained by the common tangent points as represented by the dashed lines and stars in those figures. These common tangents are obtained by solving the previous two equations for χ_c_^lh^ and χ_*c*_^hl^.

In Figure 15, we present the equilibrium isobaric phase diagram for the water-CO_2_ system. The two black dashed lines are obtained from the common-tangent method applied to the free energy densities presented in Figure 14. They represent the CO_2_ molar fraction in water in equilibrium with sI CO_2_ hydrate, χ_c_^lh^ (liquidus line), and the one in sI CO_2_ hydrate in equilibrium with water, χ_c_^hl^ (solidus line). These two lines, together with the triple equilibrium temperature *T*_eq_ (solid blue line), define the equilibrium stability region of sI CO_2_ hydrate coexisting with liquid water with dissolved CO_2_, denoted as L_*w*_ + H, in terms of temperature and CO_2_ molar fraction, χ_c_, for the given pressure. We assume that *T*_eq_ = 282.5 K for such pressure (see solid blue line). Inside the L_w_ + H region, sI CO_2_ hydrate, denoted by H, coexists with liquid water with dissolved CO_2_, denoted by L_*w*_. Hydrate exposed to water within areas of stability, with respect to pressure and temperature, and with a higher molar fraction than χ_c_^lh^, will grow. Hydrate under the same condition but exposed to water with a lower molar fraction than χ_c_^lh^ will melt. Figure 15 also shows a schematic representation of the CO_2_-in-liquid-water solubility line in a blue dash-dotted style. This line gives the CO_2_ molar fraction in liquid water in equilibrium with vapor CO_2_. A real calculation of the triple temperature and CO_2_-in liquid water solubility line requires a free energy expression for the vapor CO_2_ and is out of the scope of this work. Details on such calculations, can be found in refs (54−57, and 76). Above the triple point, the hydrate is unstable, and liquid water with dissolved CO_2_ coexist with vapor CO_2_ (L_w_ + V region). The only stable phase in the region denoted by L_w_ is liquid water with dissolved CO_2_.

### Curvature of a Flat Surface

The value 6 in the curvature expression (see eq 4 in main text) is an approximation value for the weighted summation of that expression on a flat interface. It corresponds to κ = 0 and is taken to equal one-half the sum over the entire weighted neighborhood with all voxels in a solid hydrate state^66^ (see Figure 16).

### Impact of the Initial Concentration of CO_2_ in the Water Region

In this section of the Appendix, we briefly explore the impact of the initial amount of CO_2_ in the water domain on the hydrate growth. Each voxel of the water simulation domain is started with a CO_2_ molar fraction given by χ_c_^o^ = χ + δ, where δ is a random number from a Gaussian distribution with a mean equal to zero and standard deviation σ = 10^–4^. Here, we will consider the cases, χ = 0.020, 0.025, 0.029, and 0.033.

As Figure 17 shows, the amount of hydrate covering the water region increases as a function of χ at each simulation time. For χ = 0.020 (solid black line), no hydrate growth is observed for the simulation time considered. For χ = 0.025 (red dotted line) and 0.029 (blue dotted dashed line), a diffusion-controlled growth dominates. Finally, for χ = 0.033, early on, diffusion-controlled planar crystal growth is dominant. However, the hydrate fraction suddenly increases steadily after a specific simulation time. As mentioned in the main text, this later stage corresponds to the diffusion-controlled growth of needle crystals with parabolic tips, where the growth occurs mainly at the tip region.

## References

[ref1] Sloan E. D.; Koh C. A. Clathrate Hydrates of Natural Gases, 3rd ed.; CRC Press, Taylor and Francis Group: Boca Raton, FL, 2007.

[ref2] Sloan E. D. Fundamental principles and applications of natural gas hydrates. Nature 2003, 426, 353–363. 10.1038/nature02135. 14628065

[ref3] Ripmeester J. A.; Tse J. S.; Ratcliffe C. I.; Powell B. M. A new clathrate hydrate structure. Nature 1987, 325, 135–136. 10.1038/325135a0.

[ref4] Kvenvolden K. A. Natural gas hydrate occurrence and issues. Ann. N. Y. Acad. Sci. 1994, 715, 232–246. 10.1111/j.1749-6632.1994.tb38838.x.

[ref5] Jiang L.; Xu N.; Liu Q.; Cheng Z.; Liu Y.; Zhao J. Review of Morphology Studies on Gas Hydrate Formation for Hydrate-Based Technology. Cryst. Growth Des 2020, 20, 8148–8161. 10.1021/acs.cgd.0c01331.

[ref6] Kodama T.; Ohmura R. Crystal growth of clathrate hydrate in liquid water in contact with methane+ethane+propane gasmixture. J. Chem. Technol. Biotechnol. 2014, 89, 1982–1986. 10.1002/jctb.4292.

[ref7] Chong Z. R.; Yang S. H. B.; Babu P.; Linga P.; Li X. S. Review of natural gas hydrates as an energy resource: prospects and challenges. Appl. Energy 2016, 162, 1633–1652. 10.1016/j.apenergy.2014.12.061.

[ref8] Hassanpouryouzband A.; Joonaki E.; Vasheghani Farahani M.; Takeya S.; Ruppel C.; Yang J.; English N. J.; Schicks J. M.; Edlmann K.; Mehrabian H.; Aman Z. M.; Tohidi B. Gas Hydrates in Sustainable Chemistry. Chem. Soc. Rev. 2020, 49, 5225–5309. 10.1039/C8CS00989A. 32567615

[ref9] Cannone S. F.; Lanzini A.; Santarelli M. A review on CO2 capture technologies with focus on CO2-enhanced methane recovery from hydrate. Energies 2021, 14, 387 10.3390/en14020387.

[ref10] Fournaison L.; Delahaye A.; Chatti I.; Petitet J.-P. CO_2_ Hydrates in Refrigeration Processes. Ind. Eng. Chem. Res. 2004, 43, 6521–6526. 10.1021/ie030861r.

[ref11] Khan M. N.; Peters C. J.; Koh C. A. Desalination using gas hydrates: The role of crystal nucleation, growth and separation. Desalination 2019, 468, 114049 10.1016/j.desal.2019.06.015.

[ref12] Zhang X.; Wang J.; Yang H.; Li J.; Li Y.; Wu Q. Formation and storage characteristics of CO_2_ hydrate in porous media: Effect of liquefaction amount on the formation rate, accumulation amount. Appl. Therm. Eng. 2022, 214, 118747 10.1016/j.applthermaleng.2022.118747.

[ref13] Zhang L.; Kuang Y.; Dai S.; Wang J.; Zhao J.; Song Y. Kinetic enhancement of capturing and storing greenhouse gas and volatile organic compound: Micro-mechanism and micro-structure of hydrate growth. Chem. Eng. J. 2020, 379, 122357 10.1016/j.cej.2019.122357.

[ref14] Zheng J.; Chong Z. R.; Qureshi M. F.; Linga P. Carbon Dioxide Sequestration via Gas Hydrates: A Potential Pathway toward Decarbonization. Energy Fuels 2020, 34, 10529–10546. 10.1021/acs.energyfuels.0c02309.

[ref15] Qureshi M. F.; Zheng J.; Khandelwal H.; Venkataraman P.; Usadi A.; Barckholtz T. A.; Mhadeshwar A. B.; Linga P. Laboratory demonstration of the stability of CO_2_ hydrates in deep-oceanic sediments. Chem. Eng. J. 2022, 432, 134290 10.1016/j.cej.2021.134290.

[ref16] Aman Z. M. Hydrate Risk Management in Gas Transmission Lines. Energy Fuels 2021, 35, 14265–14282. 10.1021/acs.energyfuels.1c01853.

[ref17] Majid A. A. A.; Worley J.; Koh C. C. Thermodynamic and Kinetic Promoters for Gas Hydrate Technological Applications. Energy Fuels 2021, 35, 19288–19301. 10.1021/acs.energyfuels.1c02786.

[ref18] Stoner H. M.; Phan A.; Striolo A.; Koh C. A. Water wettability coupled with film growth on realistic cyclopentane hydrate surfaces. Langmuir 2021, 37, 12447–12456. 10.1021/acs.langmuir.1c02136. 34644089

[ref19] Phan A.; Stoner H. M.; Stamatakis M.; Koh C. A.; Striolo A. Surface morphology effects on clathrate hydrate wettability. J. Colloid Interface Sci. 2022, 611, 421–431. 10.1016/j.jcis.2021.12.083. 34968961

[ref20] Uchida T.; Ebinuma T.; Kawabata J.; Narita H. Microscopic observations of formation processes of clathrate-hydrate at an interface between water and carbon dioxide. J. Cryst. Growth 1999, 204, 348–356. 10.1016/S0022-0248(99)00178-5.

[ref21] Uchida T.; Ikeda I. Y.; Takeya S.; Ebinuma T.; Nagao J.; Narita H. CO_2_ hydrate film formation at the boundary between CO_2_ and water: Effects of temperature, pressure and additives on the formation rate. J. Cryst. Growth 2002, 237-239, 383–387. 10.1016/S0022-0248(01)01822-X.

[ref22] Shigehara S.; Ohmura R. Investigation of crystal growth of CO2 hydrate in aqueous fructose solution for the potential application in carbonated solid foods. Food Chem. 2022, 371, 131369 10.1016/j.foodchem.2021.131369. 34808771

[ref23] Ohmura R.; Shimada W.; Uchida T.; Mori Y. H.; Takeya S.; Nagao J.; Minagawa H.; Ebinuma T.; Narita H. Clathrate hydrate crystal growth in liquid water saturated with a hydrate-forming substance: variations in crystal morphology. Philos. Mag. 2004, 84, 1–16. 10.1080/14786430310001623542.

[ref24] Ohmura R.; Matsuda S.; Uchida T.; Ebinuma T.; Narita H. Clathrate Hydrate Crystal Growth in Liquid Water Saturated with a Guest Substance: Observations in a Methane + Water System. Cryst. Growth Des 2005, 5, 953–957. 10.1021/cg049675u.

[ref25] Lee J. D.; Song M.; Susilo R.; Englezos P. Dynamics of Methane-Propane Clathrate Hydrate Crystal Growth from Liquid Water with or without the Presence of n-Heptane. Cryst. Growth Des 2006, 6, 1428–1439. 10.1021/cg0600647.

[ref26] Watanabe S.; Saito K.; Ohmura R. Crystal Growth of Clathrate Hydrate in Liquid Water Saturated with a Simulated Natural Gas. Cryst. Growth Des 2011, 11, 3235–3242. 10.1021/cg2005024.

[ref27] Jiang X.; Sun B.; Wang Z.; Zhou W.; Ji J.; Chen L. Methane hydrate crystal growth on shell substrate. J. Chem. Eng. 2022, 43, 50–61. 10.1016/j.cjche.2021.12.025.

[ref28] Matsuura R.; Horii S.; Alavi S.; Ohmura R. Diversity in Crystal Growth Dynamics and Crystal Morphology of Structure-H Hydrate. Cryst. Growth Des 2019, 19, 6398–6404. 10.1021/acs.cgd.9b00870.

[ref29] Saito K.; Sum A. K.; Ohmura R. Correlation of Hydrate-Film Growth Rate at the Guest/Liquid-Water Interface to Mass Transfer Resistance. Ind. Eng. Chem. Res. 2010, 49, 7102–7103. 10.1021/ie1000696.

[ref30] Martinez C.; Sandoval J. F.; Ortiz N.; Ovalle S.; Beltran J. G. Mechanisms, Growth Rates, and Morphologies of Gas Hydrates of Carbon Dioxide, Methane, and Their Mixtures. Methane 2022, 1, 2–23. 10.3390/methane1010002.

[ref31] Ohmura R.; Mori Y. H. Critical conditions for CO_2_ hydrate films to rest on submarine CO_2_ pond surfaces: A mechanistic study. Environ. Sci. Technol. 1998, 32, 1120–1127. 10.1021/es9700764.

[ref32] Li N.; Kan J.-Y.; Sun C.-Y.; Chen G.-J. Hydrate formation from liquid CO_2_ in a glass beads bed. Chin. J. Chem. Eng. 2022, 43, 185–191. 10.1016/j.cjche.2022.01.008.

[ref33] Lehmkühler F.; Paulus M.; Sternemann C.; Lietz D.; Venturini F.; Gutt C.; Tolan M. The carbon dioxide-water interface at conditions of gas hydrate formation. J. Am. Chem. Soc. 2009, 131, 585–889. 10.1021/ja806211r. 19105749

[ref34] Qureshi M. F.; Dhamu V.; Usadi A.; Barckholtz T. A.; Mhadeshwar A. B.; Linga P. CO_2_ hydrate formation kinetics and morphology observations using high-pressure liquid CO_2_ applicable to sequestration. Energy Fuels 2022, 36, 10627–10641. 10.1021/acs.energyfuels.1c03840.

[ref35] Tohidi B.; Anderson R.; Clenell M. B.; Burgass R. W.; Biderkab A. B. Visual observation of gas-hydrate formation and dissociation in synthetic porous media by means of glass micromodels. Geology 2001, 29, 867–870. 10.1130/0091-7613(2001)029<0867:VOOGHF>2.0.CO;2.

[ref36] Wang W.; Wang X.; Li Y.; Liu S.; Yao S.; Song G. Study on crystal growth and aggregated microstructure of natural gas hydrate under flow conditions. Energy 2020, 213, 118999 10.1016/j.energy.2020.118999.

[ref37] Oya S.; Aifaa M.; Ohmura R. Formation, growth and sintering of CO_2_ hydrate crystals in liquid water with continuous CO_2_ supply: Implication for subsurface CO_2_ sequestration. Int. J. Green Gas Control 2017, 63, 386–391. 10.1016/j.ijggc.2017.06.007.

[ref38] Aifaa M.; Kodama T.; Ohmura R. Crystal Growth of Clathrate Hydrate in a Flowing Liquid Water System with Methane Gas. Cryst. Growth Des 2015, 15, 559–563. 10.1021/cg500992c.

[ref39] Ovalle S.; Martinez C.; Beltran J. G. Morphology and Interphase Boundary Behavior of Gas Hydrates of Single, Binary, and Ternary Guests. Energy Fuels 2022, 36, 10512–10518. 10.1021/acs.energyfuels.2c00956.

[ref40] Ueno H.; Akiba H.; Akatsu S.; Ohmura R. Crystal growth of clathrate hydrates formed with methane+carbon dioxide mixed gas at the gas/liquid interface and in liquid water†. New J. Chem. 2015, 39, 8254 10.1039/C5NJ01080B.

[ref41] Lim Y.-A.; Babu P.; Kumar R.; Linga P. Morphology of Carbon Dioxide-Hydrogen-Cyclopentane Hydrates with or without Sodium Dodecyl Sulfate. Cryst. Growth Des 2013, 13, 2047–2059. 10.1021/cg400118p.

[ref42] Maruyama M.; Matsuura R.; Ohmura R. Crystal growth of clathrate hydrate formed with H_2_ + CO_2_ mixed gas and tetrahydropyran. Sci. Rep. 2021, 11, 11315 10.1038/s41598-021-90802-6. 34059746 PMC8167026

[ref43] Babu P.; Yee D.; Linga P.; Palmer A.; Khoo B. C.; Tan T. S.; Rangsunvigit P. Morphology of Methane Hydrate Formation in Porous Media. Energy Fuels 2013, 27, 3364–3372. 10.1021/ef4004818.

[ref44] Veluswamy H. P.; Hong Q. W.; Linga P. Morphology Study of Methane Hydrate Formation and Dissociation in the Presence of Amino Acid. Cryst. Growth Des. 2016, 16, 5932–5945. 10.1021/acs.cgd.6b00997.

[ref45] Kishimoto M.; Iijima S.; Ohmura R. Crystal Growth of Clathrate Hydrate at the Interface between Seawater and Hydrophobic-Guest Liquid: Effect of Elevated Salt Concentration. Ind. Eng. Chem. Res. 2012, 51, 5224–5229. 10.1021/ie202785z.

[ref46] Ismail N. A.; Koh C. A. Growth rate and morphology study of tetrahydrofuran hydrate single crystals and the effect of salt. CrystEngComm 2022, 24, 4301–4311. 10.1039/D2CE00176D.

[ref47] Matsuura R.; Ozawa K.; Alavi S.; Ohmura R. Crystal Growth of Clathrate Hydrate with Ozone: Implication for Ozone Preservation. ACS Sustainable Chem. Eng. 2020, 8, 15678–15684. 10.1021/acssuschemeng.0c05345.

[ref48] Ozawa K.; Ohmura R. Crystal growth of clathrate hydrate with methane plus partially Water Soluble Large-Molecule Guest Compound. Cryst. Growth Des 2019, 19, 1689–1694. 10.1021/acs.cgd.8b01625.

[ref49] Zhang Z.-C.; Wu N.-Y.; Liu C.-L.; Hao X.-L.; Zhang Y.-C.; Gao K.; Peng B.; Zheng C.; Tang W.; Guo G.-J. Molecular simulation studies on natural gas hydrate nucleation and growth: A review. China Geol. 2022, 5, 330–344. 10.31035/cg2022017.

[ref50] Das S.; Tadepalli K. M.; Roy S.; Kumar R. A review of clathrate hydrate nucleation, growth and decomposition studied using molecular dynamics simulation. J. Mol. Liq. 2022, 348, 118025 10.1016/j.molliq.2021.118025.

[ref51] Tung Y.-T.; Chen L.-J.; Chen Y.-P.; Lin S.-T. The Growth of Structure I Methane Hydrate from Molecular Dynamics Simulations. J. Phys. Chem. B 2010, 114, 10804–10813. 10.1021/jp102874s. 20669917

[ref52] Tung Y.-T.; Chen L.-J.; Chen Y.-P.; Lin S.-T. Growth of Structure I Carbon Dioxide Hydrate from Molecular Dynamics Simulations. J. Phys. Chem. C 2011, 115, 7504–7515. 10.1021/jp112205x. 20669917

[ref53] Wang P.-W.; Wu D. T.; Lin S.-T. Promotion mechanism for the growth of CO_2_ hydrate with urea using molecular dynamics simulations. Chem. Commun. 2021, 57, 5330 10.1039/D0CC06165D. 33928959

[ref54] Svandal A.; Kvamme B.ør.; Granasy L.; Pusztai T.; Buanes T.; Hove J. The phase-field theory applied to CO_2_ and CH_4_ hydrate. J. Cryst. Growth 2006, 287, 486–490. 10.1016/j.jcrysgro.2005.11.071.

[ref55] Svandal A.; Kuznetsova T.; Kvamme B. Thermodynamic properties and phase transtions in the H_2_O/CO_2_/CH_4_ system. Fluid Phase Equilib. 2006, 246, 177–184. 10.1016/j.fluid.2006.06.003. 16633655

[ref56] Tegze G.; Pusztai T.; Tóth G.; Gránásy L.; Svandal A.; Buanes T.; Kuznetsova T.; Kvamme B. Multiscale approach to CO_2_ hydrate formation in aqueous solution: Phase field theory and molecular dynamics. Nucleation and growth. J. Chem. Phys. 2006, 124, 234710 10.1063/1.2207138. 16821944

[ref57] Tegze G.; Gránásy L.; Kvamme B. Phase field modeling of CH_4_ hydrate conversion into CO_2_ hydrate in the presence of liquid CO_2_. Phys. Chem. Chem. Phys. 2007, 9, 3104–3111. 10.1039/B700423K. 17612734

[ref58] Fu X.; Cueto-Felgueroso L.; Juanes R. Nonequilibrium Thermodynamics of Hydrate Growth on a Gas-Liquid Interface. Phys. Rev. Lett. 2018, 120, 144501 10.1103/PhysRevLett.120.144501. 29694110

[ref59] Fu X.; Waite W. F.; Cueto-Felgueroso L.; Juanes R. Hydrate as an Analog ofMethane Hydrate in Geologic Systems Out of Thermodynamic Equilibrium. Geochemistry, Geophysics, Geosystems 2019, 20, 2462–2472. 10.1029/2019GC008250.

[ref60] Granasy L.; Toth G. I.; Warren J. A.; Podmaniczky F.; Tegze G.; Ratkai L.; Pusztai T. Phase-field modeling of crystal nucleation in undercooled liquids - A review. Prog. Mater. Sci. 2019, 106, 100569 10.1016/j.pmatsci.2019.05.002.

[ref61] Buanes T.; Kvamme B.; Svandal A. Computer simulation of CO_2_ hydrate growth. J. Cryst. Growth 2006, 287, 491–494. 10.1016/j.jcrysgro.2005.11.074.

[ref62] Buanes T.; Kvamme B.; Svandal A. Two approaches for modelling hydrate growth. J. Math. Chem. 2009, 46, 811–819. 10.1007/s10910-009-9551-3.

[ref63] Wolfram S. Statistical mechanics of cellular automata. Rev. Mod. Phys. 1983, 55, 601 10.1103/RevModPhys.55.601.

[ref64] Reuther K.; Rettenmayr M. Perspectives for cellular automata for the simulation of dendritic solidification – A review. Comput. Mater. Sci. 2014, 95, 213–220. 10.1016/j.commatsci.2014.07.037.

[ref65] Janssens K. G. F. An introductory review of cellular automata modeling of moving grain boundaries in polycrystalline materials. Math Comp Simul 2010, 80, 1361–1381. 10.1016/j.matcom.2009.02.011.

[ref66] Kremeyer K. Cellular Automata Investigations of Binary Solidification. J. Comput. Phys. 1998, 142, 243–262. 10.1006/jcph.1998.5926.

[ref67] Zerpa L. E.; Sloan E. D.; Sum A. K.; Koh C. A. Overview of CSMHyK: A transient hydrate formation model. J. Pet. Sci. Eng. 2012, 98, 122–129. 10.1016/j.petrol.2012.08.017.

[ref68] Liu F. P.; Li A. R.; Qing S. L.; Luo Z. D.; Ma Y. L. Formation kinetics, mechanism of CO_2_ hydrate and its applications. Renew. Sustain. Energy Rev. 2022, 159, 112221 10.1016/j.rser.2022.112221.

[ref69] Liu T.; Wu P.; Chen Z.; Li Y. Review on Carbon Dioxide Replacement of Natural Gas Hydrate: Research Progress and Perspectives. Energy Fuels 2022, 36, 7321–7336. 10.1021/acs.energyfuels.2c01292.

[ref70] Sinehbaghizadeh S.; Saptoro A.; Mohammadi A. H. CO_2_ hydrate properties and applications: A state of the art. Prog. Energy Combust. Sci. 2022, 93, 101026 10.1016/j.pecs.2022.101026.

[ref71] Mori Y. H.; Mochizuki t. Mass transport across clathrate hydrate films a capillary permeation model. Chem. Eng. Sci. 1997, 52, 3613–3616. 10.1016/S0009-2509(97)00169-3.

[ref72] Saito Y. Statistical Physics of Crystal Growth; World Scientific: Singapore, 1996.

[ref73] Sun S.; Gu L.; Yang Z.; Lin H.; Li Y. Thermophysical properties of natural gas hydrates: A review. Nat. Gas Ind. B 2022, 9, 246–263. 10.1016/j.ngib.2022.04.003.

[ref74] Pal P.; Abhishek G. S.; Karagadde S. A Monte Carlo approach to simulate dendritic microstructures during binary alloy solidification. Modelling Simul. Mater. Sci. Eng. 2020, 28, 085001 10.1088/1361-651X/abbabc.

[ref75] Walter J.-C.; Barkema G. T. An introduction to Monte Carlo methods. Physica A 2015, 418, 78–87. 10.1016/j.physa.2014.06.014.

[ref76] Kvamme B.; Tanaka H. Thermodynamic Stability of Hydrates for Ethane, Ethylene, and Carbon Dioxide. J. Phys. Chem. 1995, 99, 7114–7119. 10.1021/j100018a052.

[ref77] Liu F.; Goldenfeld N. Generic features of late-stage crystal growth. Phys. Rev. A 1990, 42, 895–903. 10.1103/PhysRevA.42.895. 9904104

[ref78] Kvamme B.; Clarke M. Hydrate phase transition kinetic modeling for nature and industry–where are we and where do we go?. Energies 2021, 14, 4149 10.3390/en14144149.

[ref79] Skovborg P.; Rasmussen P. A mass transport limited model for growth of methane and ethane gas hydrates. Chem. Eng. Sci. 1994, 49, 1131–1143. 10.1016/0009-2509(94)85085-2.

[ref80] Turner D. J.; Miller K. T.; Dendy Sloan E. Methane hydrate formation and an inward growing shell model in water-in-oil dispersions. Chem. Eng. Sci. 2009, 64, 3996–4004. 10.1016/j.ces.2009.05.051.

[ref81] Hashemi S.; Macchi A.; Servio P. Gas Hydrate Growth Model in a Semibatch Stirred Tank Reactor. Ind. Eng. Chem. Res. 2007, 46, 5907–5912. 10.1021/ie061048+.

[ref82] Kar A.; Bhati A.; Acharya P. V.; Mhadeshwar A.; Venkataraman P.; Barckholtz T. A.; Bahadur V. Diffusion-based modeling of film growth of hydrates on gas-liquid interfaces. Chem. Eng. Sci. 2021, 234, 116456 10.1016/j.ces.2021.116456.

[ref83] Abe Y.; Ma X.; Yanai T.; Yamane K. Development of formation and growth models of CO2 Hydrate Film. AIChE J. 2016, 62, 4078–4089. 10.1002/aic.15304.

[ref84] Zhao J.; Liang H.; Yang L.; Zhang X.; Song Y.; Sum A. K. Growth kinetics and gas diffusion in formation of gas hydrates from Ice. J. Phys. Chem. C 2020, 124, 12999–13007. 10.1021/acs.jpcc.0c03009.

[ref85] Liu Z.; Sun B.; Wang Z.; Chen L. New mass-transfer model for predicting hydrate film thickness at the gas-liquid interface under different Thermodynamics-hydrodynamics-saturation Conditions. J. Phys. Chem. C 2019, 123, 20838–20852. 10.1021/acs.jpcc.9b03843.

[ref86] Honda K.; Fujikawa R.; Ma X.; Yamamoto N.; Fujiwara K.; Kaneko A.; Abe Y. The formation and growth model of a CO_2_ hydrate layer based on molecular dynamics. AIChE J. 2022, 68, e17406 10.1002/aic.17406.

[ref87] Herri J.-M.; Kwaterski M. Derivation of a Langmuir type of model to describe the intrinsic growth rate of gas hydrates during crystallisation from gas mixtures. Chem. Eng. Sci. 2012, 81, 28–37. 10.1016/j.ces.2012.06.016.

[ref88] Vlasov V. A. Formation and dissociation of gas hydrate in terms of chemical kinetics. Reac. Kinet. Mech. Catal. 2013, 110, 5–13. 10.1007/s11144-013-0578-x.

[ref89] Panhuis M.; Patterson C. H.; Lynden-Bell R. M. A molecular dynamics study of carbon dioxide in water: diffusion, structure and thermodynamics. Mol. Phys. 1988, 94, 963–972. 10.1080/002689798167539.

[ref90] Zhao G.; Gong G.; Sun H.; Chen B.; Zheng J.-N.; Yang M. Effect of Methane Solubility on Hydrate Formation and Dissociation: Review and Perspectives. Energy Fuels 2022, 36, 7269–7283. 10.1021/acs.energyfuels.2c01017.

[ref91] Servio P.; Englezos P. Effect of temperature and pressure on the solubility of carbon dioxide in water in the presence of gas hydrate. Fluid Phase Equilib. 2001, 190, 127–134. 10.1016/S0378-3812(01)00598-2.

[ref92] Mullins W. W.; Sekerka R. F. Stability of a planar interface during solidification of a dilute binary alloy. J. Appl. Phys. 1964, 35, 444 10.1063/1.1713333.

[ref93] Adamova T. P.; Stoporev A. S.; Manakov A. Y. Visual studies of methane hydrate formation on the water-oil boundaries. Cryst. Growth Des 2018, 18, 6713–6722. 10.1021/acs.cgd.8b00986.

[ref94] Jin Y.; Nagao J. Morphological change in structure H clathrates of methane and liquid hydrocarbon at the liquid-liquid interface. Cryst. Growth Des 2011, 11, 3149–3152. 10.1021/cg2003996.

[ref95] Svandal A.; Kuznetsova T.; Kvamme B. Thermodynamic properties and phase transitions in the H_2_O/CO_2_/CH_4_ system. Phys. Chem. Chem. Phys. 2006, 8, 1707–1713. 10.1039/b516375g. 16633655

[ref96] Servio P.; Englezos P. Morphology of methane and carbon dioxide hydrates formed from water droplets. AIChE J. 2003, 49, 269–276. 10.1002/aic.690490125.

[ref97] Strukov D. A.; Adamova T. P.; Manakov A. Y. Nucleation and growth of methane and carbon dioxide hydrates on wetting liquid films. Cryst. Growth Des 2023, 23, 354–361. 10.1021/acs.cgd.2c01048.

